# Deep-seated psychological histories of COVID-19 vaccine hesitance and resistance

**DOI:** 10.1093/pnasnexus/pgac034

**Published:** 2022-03-24

**Authors:** Terrie E. Moffitt, Avshalom Caspi, Antony Ambler, Kyle Bourassa, HonaLee Harrington, Sean Hogan, Renate Houts, Sandhya Ramrakha, Stacy L. Wood, Richie Poulton

**Affiliations:** 1 Department of Psychology and Neuroscience, Duke University, Durham, NC, USA; 2 Social, Genetic, and Developmental Psychiatry Centre, Institute of Psychiatry, Psychology and Neuroscience, King’s College London, London, UK; 3 Center for the Study of Population Health & Aging, Duke University Population Research Institute, Durham, NC, USA.; 4 Geriatric Research, Education, and Clinical Center, VA Durham Healthcare System, Durham, NC, USA; 5 Department of Psychology and Dunedin Multidisciplinary Health and Development Research Unit, University of Otago, Dunedin, NZ; 6 Consumer Innovation Consortium, Department of Business Management, North Carolina State University, Raleigh, NC, USA

**Keywords:** Vaccine resistance, vaccine hesitance, COVID-19, health policy, health education

## Abstract

To design effective pro-vaccination messaging, it is important to know “where people are coming from”—the personal experiences and long-standing values, motives, lifestyles, preferences, emotional tendencies, and information-processing capacities of people who end up resistant or hesitant toward vaccination. We used prospective data from a 5-decade cohort study spanning childhood to midlife to construct comprehensive early-life psychological histories of groups who differed in their vaccine intentions in months just before COVID vaccines became available in their country. Vaccine-resistant and vaccine-hesitant participants had histories of adverse childhood experiences that foster mistrust, longstanding mental-health problems that foster misinterpretation of messaging, and early-emerging personality traits including tendencies toward extreme negative emotions, shutting down mentally under stress, nonconformism, and fatalism about health. Many vaccine-resistant and -hesitant participants had cognitive difficulties in comprehending health information. Findings held after control for socioeconomic origins. Vaccine intentions are not short-term isolated misunderstandings. They are part of a person’s style of interpreting information and making decisions that is laid down before secondary school age. Findings suggest ways to tailor vaccine messaging for hesitant and resistant groups. To prepare for future pandemics, education about viruses and vaccines before or during secondary schooling could reduce citizens’ level of uncertainty during a pandemic, and provide people with pre-existing knowledge frameworks that prevent extreme emotional distress reactions and enhance receptivity to health messages. Enhanced medical technology and economic resilience are important for pandemic preparedness, but a prepared public who understands the need to mask, social distance, and vaccinate will also be important.

## Introduction.

With COVID-19, the world experienced a death-causing infectious pandemic during 2020 and 2021. Vaccines became available starting in December 2020 and saved lives. Yet country by country, when COVID-19 vaccines have been rolled out, a significant proportion of the population has not taken the opportunity to be vaccinated. Unfortunately, slow vaccine acceptance delays achievement of the public-health goal of preventing virus transmission. Even if a majority of developed nations’ eligible citizens are eventually vaccinated by 2022, vaccine intentions will remain a concern because most of the world population is still unvaccinated, parents must decide whether to vaccinate children, and already-vaccinated citizens will need booster vaccinations. Moreover, after most eligible citizens accept vaccination, a minority retain their initial resistant stance toward COVID-19 vaccination. Institutions have several means to motivate vaccination (e.g., employer mandates, financial incentives), but effective public health messaging remains an essential tool to overcome hesitancy and resistance.

In this context, scientific interest in vaccination intentions has intensified. Most studies have necessarily taken a “point-in-time” survey approach to determine what concurrent characteristics of a person (e.g., political identity, age, income) are correlated with different vaccine intentions. To add new insights, we report a mid-2021 survey of vaccine intentions among the members of a 5-decade longitudinal birth cohort study, the Dunedin Study. Embedding data collection about COVID-vaccine intentions in an ongoing longitudinal study allowed us to harness a lifetime archive of rich prospective data to uncover personal psychological histories associated with vaccine intentions. These data allowed us to test whether factors underlying slow adoption of the COVID-19 vaccine are longstanding characteristics that emerge early in life. By studying deep roots of vaccine intentions, we aimed to provide new insights for public health messaging that is more empathic, respectful, and sensitive to the deep-seated needs of vaccine-hesitant and resistant audiences.

Early rapid-response cross-sectional surveys provided valuable information about respondents’ COVID-19 vaccine intentions and about proximal reasons that respondents endorse for the intentions they hold, such as reasonable concerns about the safety of rapidly developed vaccines (e.g., Cascini et al. 2021; Freeman et al. 2021; Paul et al. 2021; Tibbetts 2021). Based on accumulating evidence, various strategies have been adapted from the marketplace to persuade people who are not vaccinated to get vaccinated: tackle misinformation, use trusted messengers, trigger fear of missing out, anticipate regret if a love-done died, explain how the vaccine works, use influencers, report trends in rising numbers who have been vaccinated, leverage the scarcity and preciousness of the vaccine, or nudge people to overcome apathy by sending reminders (Dai et al. 2021; Milkman et al. 2021; NASEM 2021; Nuffield Council on Bioethics, 2021; Tibbetts, 2021; Volpp et al 2021; Wood et al. 2021; Wood & Schulman 2021). Many of these tactics have proven effective; however, the level of success varies greatly (Nuti & Armstrong, 2021). One reason for this variation may be that many strategies are ‘one-size-fits-all here-and-now’ approaches. They apply social persuasion processes or deliver information about benefits of vaccination, both enacted proximal to the time of vaccine decision-making (Brewer et al. 2017). Unfortunately, giving people information about health benefits generally shows modest success in achieving health-behavior change (Nuti & Armstrong, 2021). Moreover, after health-messaging strategies, nudges, and mandates have persuaded the majority to get vaccinated, a significant portion of the population remains vaccine-resistant.

Accordingly, public health and consumer researchers frequently call for tailored vaccination messages that respond to deep personal identities, feelings, and core beliefs (Hunter et al. 2021; Lewis and Oyserman, 2016; Sgaier 2021; Tufekci 2021; Wood 2012; Wood and Schulman, 2021). For many people, emotion rather than facts drives health decisions (Van Bavel et al. 2020), a phenomenon that argues “You can’t reason somebody out of something they were not reasoned into in the first place” (Sapolsky, 2021). There is also evidence that vaccine hesitancy may be associated with lower pre-pandemic levels of cognitive capacities needed to process facts (Batty et al. 2021). To design effective pro-vaccination messaging, it is important to know “where people are coming from”—the personal experiences and long-standing values, motives, lifestyles, preferences, emotional tendencies, and information-processing capacities of people who end up hesitant or resistant toward vaccination. Prior American surveys report that un-vaccinated individuals tend to be, for example, Republicans, Southerners, or lacking education (Cascini et al. 2021; Volpp et al 2021). But there is huge variation within such demographic groups, and demographic groups are poor proxies for people’s actual long-held personal beliefs, preferences, cognitive abilities, and motivations that might feed into their vaccine intentions (Freeman et al. 2021). Valuable point-in-time psychological profiles of vaccine intentions have been reported (Murphy et al 2021). However, psychologically rich prospective data about the early-life origins of vaccine intentions, which are unavailable in point-in-time surveys, are needed to fill this knowledge gap.

To help fill this gap, in mid-2021 we conducted a survey of vaccine intentions among a birth cohort of 49-year-old adults born in 1972–73 in New Zealand: the Dunedin Study (Poulton et al. 2015). Briefly, the cohort is primarily white (93% self-identified), and matches its nation’s levels on educational attainment, and key health indicators (e.g., body mass index, smoking, physical activity, and physician visits). Data have been collected at birth and each participant came to the research unit for private interviews and examinations at ages 3, 5, 7, 9, 11, 13, 15, 18, 21, 26, 32, 38, and, most recently at age 45 years, when 94% of Study members still alive in 2019 participated. In April-July 2021, we invited the 942 living Study members residing in New Zealand and Australia to report their vaccine intentions in a rapid survey, obtaining an 88% response rate (N=832). Responding and non-responding Study members did not differ on their childhood social class origins, childhood IQ, or history of mental disorder symptoms (Supplemental Information). Study members reported whether they intended to be vaccinated (hereafter termed “willing”), did not intend to be vaccinated (termed “resistant”), or did not know enough to decide (termed “hesitant/undecided”). As shown in Table 1, we linked the Dunedin cohort members’ 2021 vaccine intentions to archived longitudinal data in order to test hypotheses about psychological histories of COVID-19 vaccine hesitance and resistance.

We surveyed participants’ intentions in mid-2021, after access to ample information about the COVID-19 vaccine and immediately prior to the pre-announced vaccination roll-out to the general population in New Zealand and Australia in August, when they would soon be acting on their intentions. We took advantage of evidence that uptake of any new medical technology shifts over time along an S-shaped pattern. Acceptance is slow at first when a few people are willing early adopters. Acceptance then increases quickly as initially hesitant individuals gain information about the technology and shift to the willing group, before acceptances then slow again leaving a few innovation-resistant people (Rogers, 2003; Szilagyi et al. 2021; Wood and Schulman, 2021). From late 2020 our participants had the benefit of extensive news-media and social-media coverage of vaccine roll-outs in other countries (e.g., Britain, Israel, the USA), including media reports that locales with higher vaccine uptake had lower death rates. Thus, timing of the survey toward the end of the acceptance S-curve helped to identify groups most in need of tailored health messaging. Up to the time of the survey, New Zealand maintained a zero-COVID policy, recording only 22 deaths.

## Results.

Cohort members reported in April-July 2021 whether they: (a) definitely or probably intended to be vaccinated (N=622 [75%], 50% female; hereafter termed “Vaccine-Willing”); (b) did not know enough to decide (N=101 [12%], 56% female; termed “Vaccine-Hesitant/Undecided”); or (c) definitely or probably did not intend to be vaccinated (N=109 [13%], 49% female; termed “Vaccine-Resistant”). We used Ordinary Least Squares regression to compare the Vaccine-Resistant and Vaccine-Hesitant groups against the Vaccine-Willing. All regression coefficients are standardized coefficients that can be interpreted as effect sizes in standard deviation units, and that can be compared across variables (see Supplemental Information).

### Group demographics.

**Figure 1.**
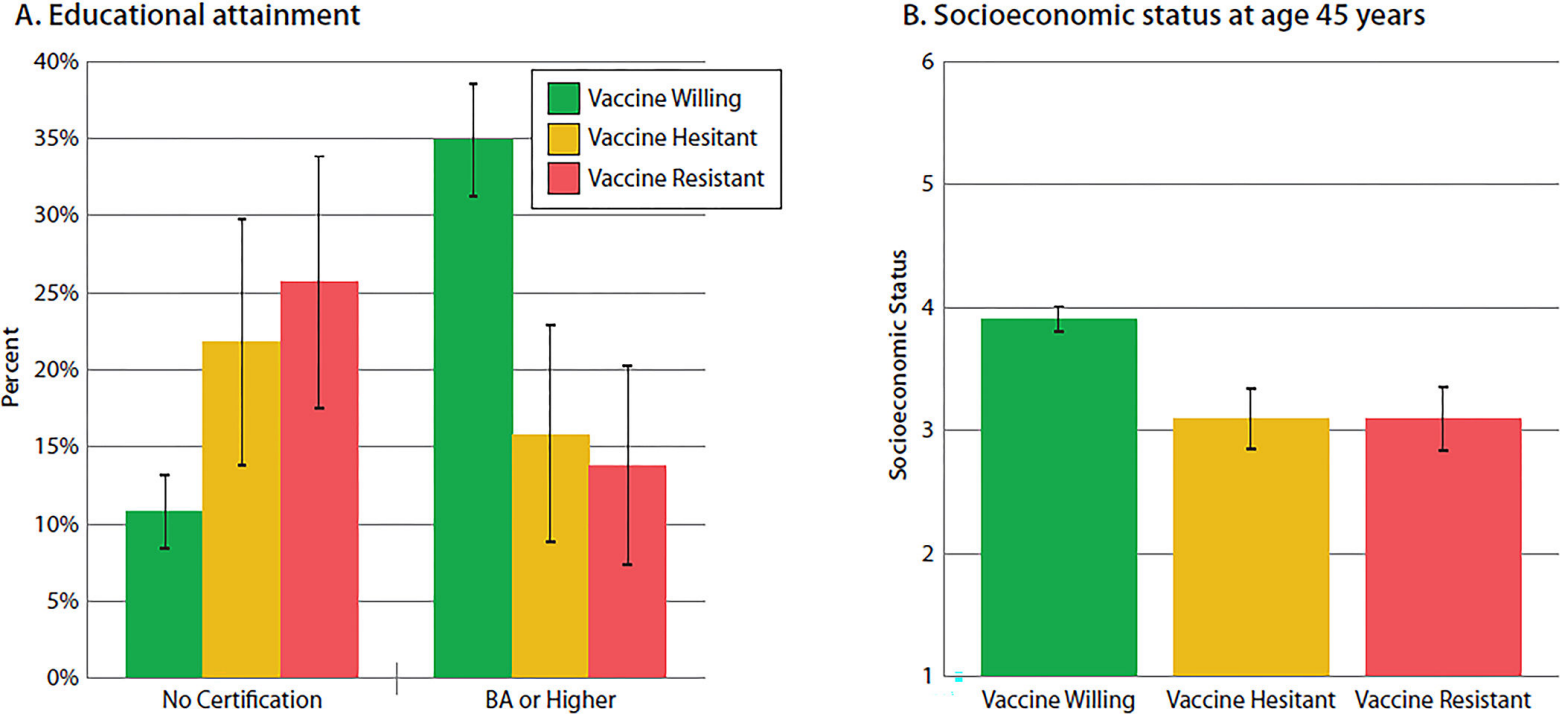
Educational attainment and socioeconomic status among the Vaccine-Willing, Vaccine-Hesitant and Vaccine-Resistant groups. Figure presents (**A**) the percentage leaving school with no certification and the percentage with a BA or higher and (**B**) the mean socioeconomic status of the Vaccine-Willing (green), Vaccine-Hesitant (yellow) and Vaccine-Resistant (red) groups. Mean proportions are sex-adjusted. Error bars represent 95% Confidence Intervals, to visualize if the confidence intervals between the groups overlap. Statistical tests are reported in Supplemental Information Table S2.

As in other developed countries, vaccine intentions followed the expected social gradient. Truncated education (Figure 1A) and lower socioeconomic status (Figure 1B) distinguished the Vaccine-Resistant and Vaccine-Hesitant groups from the Vaccine-Willing. For example, approximately one-quarter of the Vaccine-Resistant and -Hesitant groups had left high school without any qualification whereas only one-tenth of the Vaccine-Willing did so. In contrast, 35% of the Vaccine-Willing completed university, but only about 15% of the Vaccine-Resistant and -Hesitant did so.

In this cohort, lack of experience with health-care did not notably distinguish the groups. All participants had access to universal health care. The vast majority of Willing (98%), Hesitant (99%), and Resistant (91%) Study members could identify their GP when interviewed in 2019, and they had accessed health care during the past year in broadly similar numbers (60% of the Willing, 58% of Hesitant, and 53% of Resistant).

### Reasons for and against vaccination.

**Figure 2.**
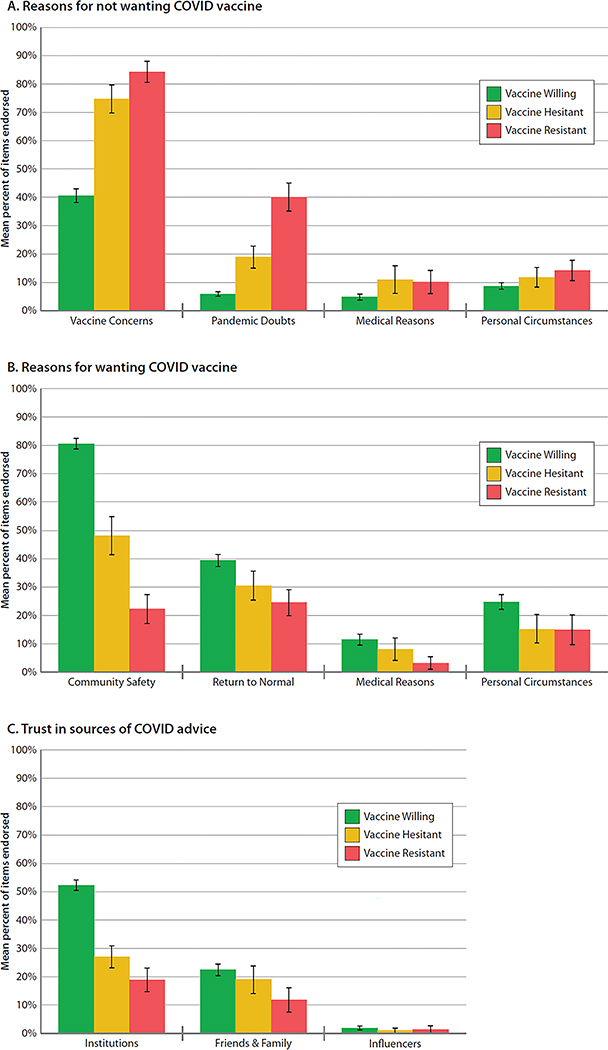
Reasons against vaccination, reasons for vaccination, and sources of trust during the COVID-19 pandemic. Figure presents the mean proportion of items endorsed among the Vaccine-Willing (green), Vaccine-Hesitant (yellow) and Vaccine-Resistant (red) groups. Mean proportions are sex-adjusted. Error bars represent 95% Confidence Intervals, to visualize if the confidence intervals between the groups overlap. Statistical tests are reported in Supplemental Information Table S2.

All Study members answered questions about why they would want (13 items) or would not want (20 items) to be vaccinated (Supplemental Information). Principal component analysis of the item responses yielded four scales capturing reasons against vaccination: Pandemic Doubts (e.g., Don’t believe the COVID-19 pandemic is as bad as people say it is); Vaccine Safety Concerns (e.g., We don’t know about the vaccine’s long-term effects); Medical Reasons (e.g., I’m allergic to vaccines), and Reasons It’s Not for Me (e.g., I don’t like needles). Principal component analysis also yielded four scales capturing reasons for vaccination: to ensure Community Safety (e.g., Me getting vaccinated will keep my family and friends safe); to Return to Normal (e.g., Vaccine card will allow me to travel, work, or enter businesses); Medical Reasons (e.g., I have a health problem that increases my risk of dying from COVID-19); and Personal Employment Circumstances (e.g., My job places me at high risk for COVID-19). Compared to the Vaccine-Willing group, the Vaccine-Resistant group harbored safety concerns about vaccines (B=1.27, 95% CI [1.13,1. 41], *P* < 0.001) and doubts about the pandemic (B=1.78 [1.51,2.05], *P* < 0.001) (Figure 2A). The Resistant group did not think that vaccines would improve community safety (B=−1.74 [−1.90,−1.57], *P* < 0.001) nor hasten return to normalcy (B=−.52 [−0.71,−0.35], *P* < 0.001) (Figure 2B). The Vaccine-Hesitant group tended to share these views, but to a less striking extent (Figure 2A,B). Irrespective of their vaccine intentions, few Study members cited medical reasons or personal circumstances as explanations for either wanting or not wanting a vaccine (Figure 2A,2B).

### Trusted COVID-19 information sources.

Study members also reported who they trusted for information about COVID-19 (11 items, Supplemental Information) (Figure 2C). Principal component analysis of the responses yielded three scales: Trust in Institutions (government, scientists, doctors, news media, and drug companies); Trust in Friends & Family (friends, family, co-workers, faith leaders); Trust in Influencers (celebrities, social media). Both the Vaccine-Resistant and Vaccine-Hesitant groups expressed little trust in institutions (B=−1.19 [−1.37,−1.02.], *P* < 0.001 and B=−.91 [−1.07,−0.75], *P* < 0.001, respectively). Virtually none of the 49-year-old Study members said they trusted influencers. What distinguished the Vaccine-Resistant group was that their mistrust was widespread, extending not only to institutions and influencers, but also to family, friends, and co-workers (B=−.38 [−0.56,−0.21], *P* < 0.001).

### Psychological histories.

**Figure 3.**
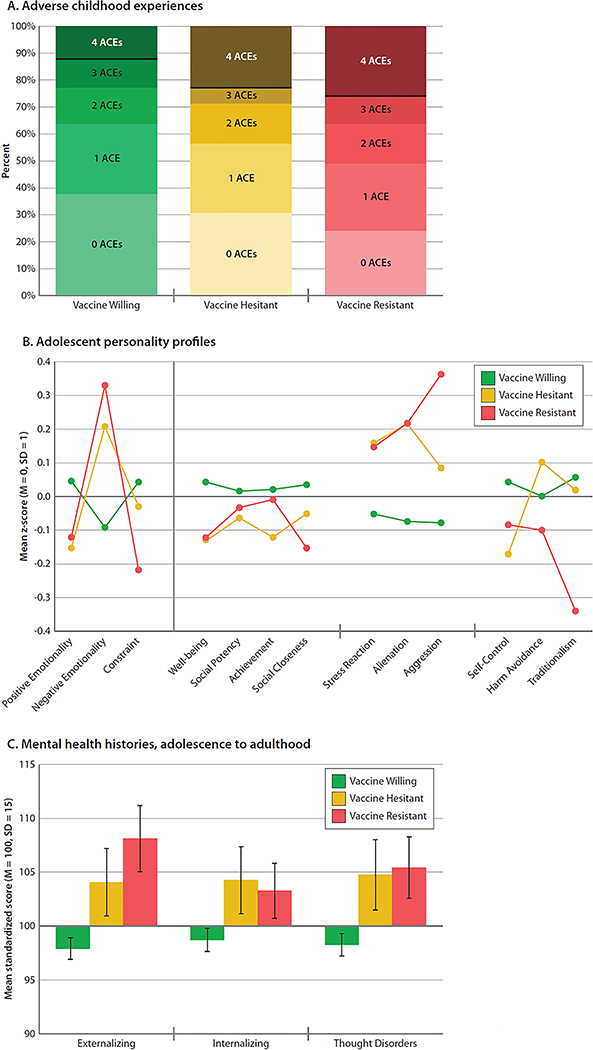
Childhood experiences, adolescent personality profiles, and mental-health histories of the Vaccine-Willing, Vaccine-Hesitant, and Vaccine-Resistant groups. Figure shows (**A**) number of Adverse Childhood Experiences (ACEs), with a black line representing the Centers for Disease Control (CDC) cut-off of clinical concern, (**B**) mean MPQ personality profiles at age 18 years, and (**C**) mean mental health symptom histories from age 18 to 45 years among the Vaccine-Willing (green), Vaccine-Hesitant (yellow), and Vaccine-Resistant (red) groups. Means are sex-adjusted. Error bars represent 95% Confidence Intervals, to visualize if the confidence intervals between the groups overlap. Statistical tests are reported in Supplemental Information Table S3.

Adult vaccine intentions harkened back to childhood experiences and adolescent personality. Forty years ago, Vaccine-Resistant (B=.44 [0.19,0.66], *P* < 0.001), and to a lesser extent Vaccine-Hesitant adults (B=.21 [−0.01,0.43], *P* = 0.052), were exposed to significantly more Adverse Childhood Experiences (ACEs), marked by abuse, neglect, threat, and deprivation (Figure 3A). Their personality profiles, assessed 30 years ago, when they were 18 years old, reveal a nuanced picture of their future intentions (Figure 3B). Of interest, Willing, Hesitant, and Resistant adults had not differed greatly from each other as adolescents in their Positive Emotionality; they scored similarly on measures of Well Being and Social Closeness (the capacity to experience joy, pleasure, and affection for others) and Social Potency and Achievement (the tendency to active and rewarding engagement in social and work environments). Instead, where the Vaccine-Resistant and Vaccine-Hesitant groups differed from the Vaccine-Willing was in their adolescent Negative Emotionality: they scored higher on measures of Stress Reactivity (the tendency to mentally shut down under stress) (B=.21 [0.02,0.40], *P* = 0.03 and B=.19 [−0.01,0.40], *P* = 0.08, respectively), Alienation (the tendency to feel mistreated and expect the worst from others) (B=.29 [0.09,0.49], *P* = 0.006 and B=.31 [0.09,0.53], *P* = 0.005, respectively), and Aggression (the willingness to hurt others for own advantage), on which the Resistant group scored extremely high (B=.43 [0.25,0.61], *P* < 0.001 and B=.22 [0.02,0.41], *P* = 0.02, respectively). In addition, the Vaccine-Resistant group scored extremely low on Traditionalism, where a low score indicates valuing personal freedom over social norms, and being nonconformist (B=−.40 [−0.62,−0.17], *P* = 0.001). However, the Hesitant group was not as extremely high on Aggression or low on Traditionalism as the Resistant group. In secondary analyses (Supplementary Figure S1), these personality self-reports by Study members were confirmed by reports from informants who knew them well. Informants reported on the Big-Five personality scales that Resistant and Hesitant groups tended to be disagreeable and to experience extreme negative emotions.

Adult vaccine intentions were also linked to a history of mental health problems, dating back to adolescence. The mental-disorder life-histories of Vaccine-Resistant and Vaccine-Hesitant Study members show that over three decades they had experienced more symptoms of Externalizing Disorders (inattention, antisocial, substance misuse) (B=.67 [0.46,0.89], *P* < 0.001 and B=.44 [0.20,0.65], *P* < 0.001, respectively), Internalizing Disorders (anxiety, depression) (B=.31 [0.14,0.50], *P* = 0.001 and B=.34 [0.13,0.57], *P* = 0.002, respectively), and Thought Disorders (delusional beliefs, hallucinations, obsessions, compulsions) (B=.48 [0.28,0.68], *P* < 0.001 and B=.43 [0.19,0.66], *P* < 0.001, respectively), all symptoms which might interfere with receipt of health messaging, healthy decision-making, and resistance to conspiracy theories (Figure 3C). Secondary analyses of diagnosed mental disorders recorded from adolescence to age 45 showed that compared to Vaccine-Willing Study members, Resistant and Hesitant Study members’ histories tended to include a greater variety of different diagnosed mental disorders (B=.61 [0.39,0.84], *P* < 0.001 and B=.50 [0.26,0.74], *P* < 0.001, respectively), a younger age at first mental-disorder onset (B=−.34 [−0.51,−0.16], *P* < 0.001 and B=−.25 [−0.43,−0.04], *P* = 0.01, respectively), and more years of persistence of mental disorders (B=.37 [0.17,0.56], *P* < 0.001 and B=.41 [0.18,0.64], *P* < 0.001, respectively).

**Figure 4.**
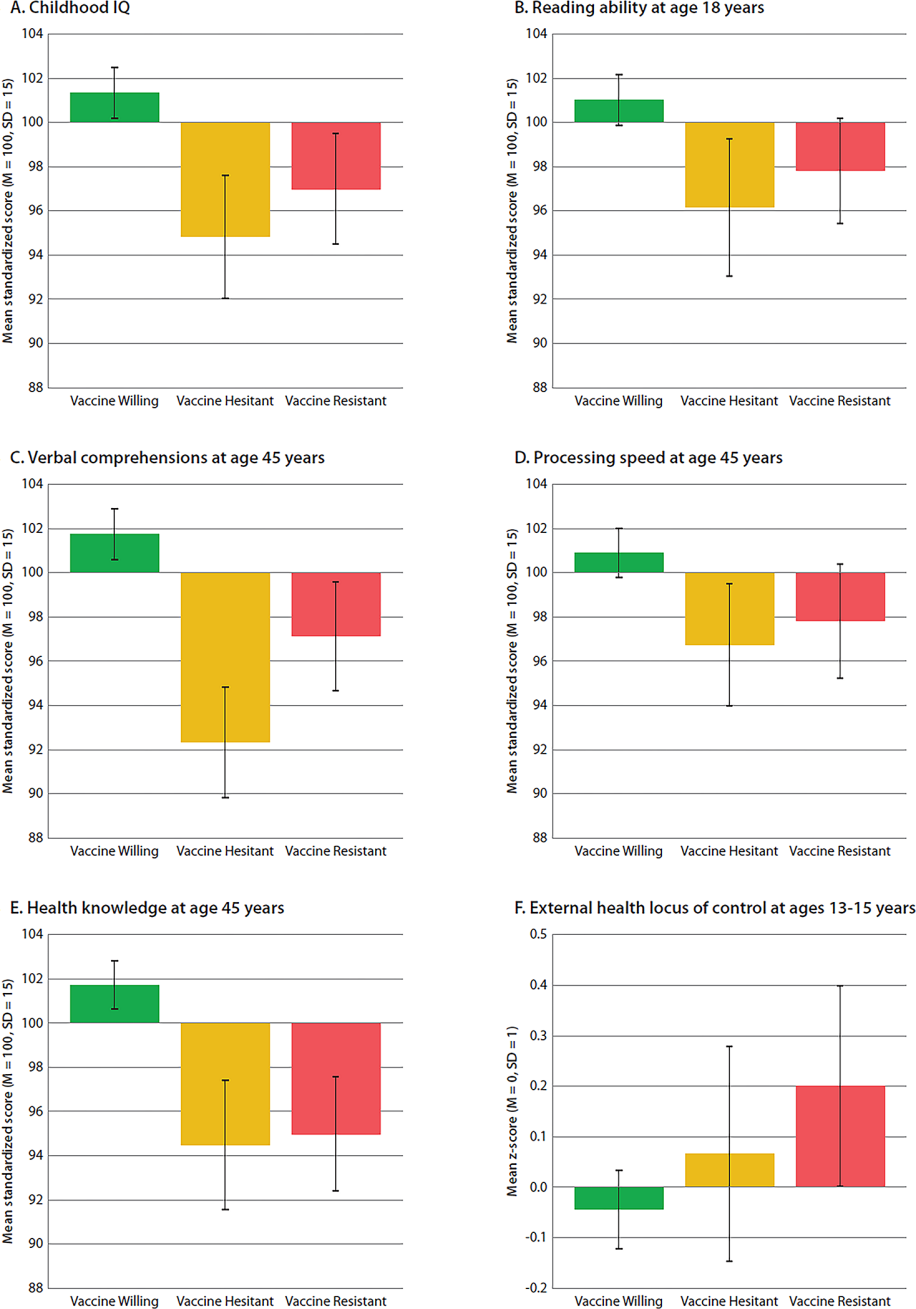
Cognitive characteristics of the Vaccine-Willing, Vaccine-Hesitant, and Vaccine-Resistant groups. Figure shows mean (**A**) childhood IQ, (**B**) reading ability at age 18 years, (**C**) verbal comprehension at age 45 years, (**D**) processing speed at age 45 years, (**E**) health knowledge at age 45 years and (**F**) health locus of control at ages 13–15 years among the Vaccine Willing (green), Vaccine Hesitant (yellow), and Vaccine Resistant (red). Means are sex-adjusted. Error bars represent 95% Confidence Intervals, to visualize if the confidence intervals between the groups overlap. Statistical tests are reported in Supplemental Information Table S4.

Adult vaccine intentions were linked to cognitive difficulties present from early life. Vaccine-Resistant and especially Vaccine-Hesitant groups performed less well on IQ tests as children (B=−.29 [−0.48,−0.−10], *P* = 0.002 and B=−.43 [−0.62,−0.22], *P* < 0.001, respectively) (Figure 4A) and were below-average readers at the time they left high school (B=−.21. [−0.39,−0.03], *P* = 0.02 and B=−.34 [−0.56,−0.12], *P* = 0.002, respectively) (Figure 4B). As adults, they had lower scores on tests of Verbal Comprehension--indicating they were less adept in thinking with language (B=−.31 [−0.48,−0.12], *P* = 0.001 and B=−.62 [−0.80,−0.43], *P* < 0.001, respectively)--but the Hesitant group scored especially low on verbal abilities. Both groups had lower scores on tests of Processing Speed, indicating that they think less efficiently (B=−.20 [−0.39,−0.00], *P* = 0.05 and B=−.31 [−0.51, −0.11], *P* = 0.004, respectively) (Figures 4C,4D).

Three years ago, when Study members were 45 years old and prior to the pandemic, we assessed their Practical Health Knowledge (Figure 4E). This measure does not depend on reading ability; it assesses understanding of basic health principles in an open-ended interview (e.g., “What are some of the reasons it is important to get your blood pressure checked?” or “What are some of the reasons you should know your family medical history?”). Both Vaccine-Resistant and Vaccine-Hesitant groups had less practical everyday health knowledge (B=−.45 [−0.62,−0.25], *P* < 0.001 and B=−.50 [−0.70,−0.29], *P* < 0.001, respectively) than the Willing group, suggesting that both groups were less prepared to manage health decisions going into the pandemic. Health Locus of Control assessed at ages 13–15 revealed that as young as adolescence, the Vaccine-Resistant group tended to believe that their health is principally due to external factors beyond their control, a matter of fate (B=.24 [0.03,0.46], *P* = 0.03) (Figure 4F).

Secondary analyses are reported in the Supplement. First, associations between each psychological history measure and the reasons against vaccination are reported in Supplementary Figure S2. “Vaccine concerns” and “doubts about the pandemic” were associated with histories of adverse childhood experiences, negative personality styles, mental health problems, cognitive difficulties, and poor health knowledge. Second, the deep-seated psychological histories associated with vaccine intentions were not simply attributable to the socioeconomic background in which hesitant and resistant individuals grew up. When we controlled for the participants’ socioeconomic background, we found that childhood adversity, tendencies toward extreme negative emotions and shutting down mentally under stress, mental health history, and cognitive difficulties continued to distinguish Vaccine-Hesitant and Vaccine-Resistant groups (Supplementary Table S5). As one example, the association between IQ scores and vaccine intentions was the same across low, medium, and high social classes, shown in Supplementary Figure S3. Third, we singled out college-educated Vaccine-Resistant individuals’ (n=15; only 13% of Resistors) to check if they differed from the less-educated majority of Vaccine-Resistant individuals on key predictors. Supplementary Figure S4 shows college-educated Resistors scored less extremely than the less-educated Resistors. This means that our findings slightly under-estimate associations between predictors and vaccine resistance for the majority of Resistors (i.e, those who have less than a college education).

## Discussion.

**Figure 5.**
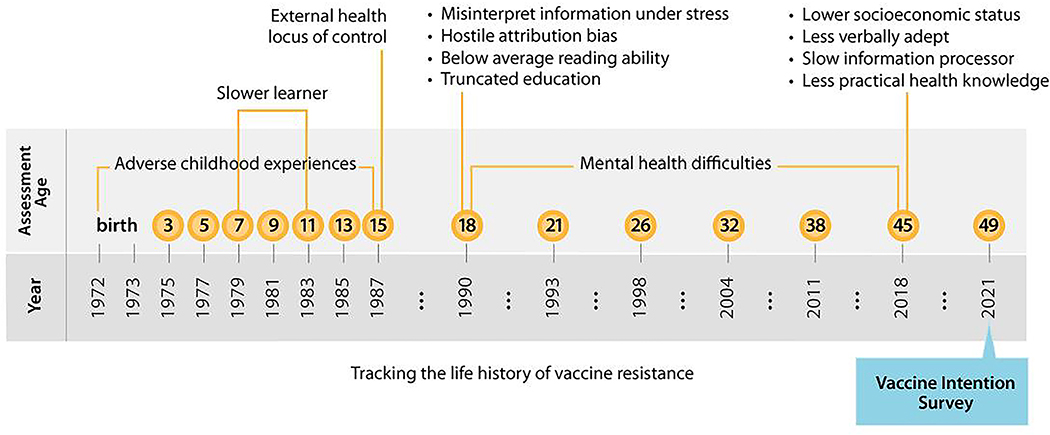
Tracking the life history of vaccine resistance. The figure charts the history of the Dunedin Study, and the deep-seated psychological histories of COVID-19 vaccine resistance.

To develop persuasive pro-vaccination messaging, it is important to know where people are coming from, especially people who end up resistant or hesitant regarding vaccination. We invited members of a 5-decade population-representative longitudinal birth-cohort study to report their vaccine intentions in the months just before COVID-19 vaccines became available in their country. We then harnessed the Study’s prospective archival data spanning childhood to midlife to describe the psychological histories of Study members who reported Vaccine-Hesitant and Vaccine-Resistant intentions, comparing them to study members who were willing to be vaccinated. The resulting personal-history findings contribute new knowledge by revealing that vaccine hesitancy and resistance can have deep roots from early life; hesitancy and resistance are not merely contemporary misunderstandings made by uninformed adults. Many Vaccine-Resistant and, to a lesser extent, Vaccine-Hesitant adults had childhood histories of adverse experiences in their families, such as abuse, mistreatment, deprivation, and neglect, that can understandably leave survivors with a lifelong legacy of mistrust. Dating back to adolescence, many had experienced chronic mental-health conditions that can foster apathy and avoidance, derail healthy decision-making, and even promote susceptibility to conspiracy theories. Personality profiles showed that, already 30 years ago as teenagers, the Vaccine-Resistant and Hesitant groups described themselves as having a propensity to reject incoming information when under stress and to misinterpret health messaging with a hostile attribution bias. Resistant and Hesitant groups were vulnerable to extreme emotions of fear and anger, they tended to shut down mentally under stress, they described themselves as non-conformists who value personal freedoms over social norms, and they felt fatalistic about their health; all tendencies that had been recorded during adolescence and confirmed by friends and family informants who knew them well. Exacerbating matters, the Hesitant and Resistant groups’ early-life personal adversities and emotional vulnerabilities were accompanied by long-standing problems with cognitive information-processing. Such cognitive deficits make it difficult for anyone to comprehend incoming health information under calm conditions, but comprehension deficits combined with extreme emotions can lead to decisions that seem inexplicable to health professionals. COVID-19 vaccine intentions can be part of a lifelong psychological style that becomes more understandable when the childhood and adolescent history behind it is appreciated (Figure 5).

In general, we found that Vaccine-Hesitant participants’ psychological histories were similar to Vaccine-Resistant participants in some areas, but in such areas Hesitant participants’ histories were on average less extreme. Relative to the Resistant group, the Hesitant group reported somewhat fewer doubts about the validity of the pandemic, expressed relatively more trust in institutions such as government, scientists, doctors, news media, and drug companies, and also endorsed more reasons to get vaccinated to preserve the safety of their community. Consistent with this pattern, on personality assessments the Hesitant group was less willing than the Resistant group to cause others discomfort, reported a less aggressive stance toward people, and was less likely to disparage social norms and rules. However, the Hesitance was not just a milder version of the Resistance. For example, relative to the Resistant group, as adults the Hesitant group had weaker verbal comprehension abilities, which probably interfered with their ability to understand complex and constantly changing information about the COVID-19 vaccines, leaving them feeling hesitant. This pattern suggests that clear and simple messaging tailored to a modest level of verbal complexity may reach the vaccine-hesitant.

In contrast to the Hesitant and Willing groups, the Vaccine-Resistant group’s mistrust was ubiquitous, extending beyond institutions and influencers, to mistrust of family, friends, and co-workers. This pattern suggests that messaging that breaks through deeply-seated extreme mistrust will be required to reach the vaccine-resistant. Clear and simple wording alone will not suffice. A better understanding of the ‘priors’ and information processing style of vaccine-resisters is urgently needed to guide new ways of engaging with this group. It further suggests why Vaccine-Resistant individuals succumb to misinformation generated by anti-vaccination messagers. Pro-vaccination health messaging does not operate in a vacuum, it must compete against powerful anti-vaccination messaging, which often reinforces themes of suspicion, mistrust, fear, anger, alienation, and conspiracy, sensationalizes fear of rare side effects, lionizes anti-establishment nonconformism, praises going against the ‘vaccinated herd’, and presents vaccination as a personal choice that must be exercised to preempt exploitation by the government. Such themes align with and reinforce the Resistant group’s longstanding beliefs that they can expect to be mistreated, victimized, and betrayed, are powerless to prevent this, and must respond to this unfairness with vigorous resistance against social norms (Browne et al. 2015; Uscinski and Parent, 2014).

This study offered certain advantages over the first wave of point-in-time COVID-19 vaccine-intention surveys. First, the cohort represents the full range of variation in its national population with no healthy volunteer bias. Thus, the data represent groups who do not respond to typical vaccine-intention surveys, including individuals with poor literacy and vaccine-resistant individuals who deeply distrust survey research. Second, this cohort has been assessed in repeated day-long clinic visits since childhood, most recently in 2019 at age 45 before the pandemic began, enabling us to study prospective psychological measures unbiased by recall failure or knowledge of participants’ 2021 vaccine-intentions. Third, over decades cohort members have learned to trust the Study’s strict confidentiality guarantee. As such, this cohort is unusually forthcoming about private lifestyles, motives, and values, including vaccine intentions. Fourth, we were able to ask cohort members about their vaccine intentions immediately before the general population got access to vaccination, but after participants had the benefit of extensive media coverage of vaccine roll-outs in other countries. This meant that participants’ intentions were not initial mild impressions, enabling us to identify groups whose intentions were informed and near-final, those most in need of persuasion to get vaccinated. Fifth, findings remained despite control for socioeconomic origins. Despite such advantages, our study has limitations. First, vaccine intentions were ascertained through self-report; eventual behavior can differ from self-reported intentions. Second, important determinants of vaccine uptake were not studied, including ease of access to vaccination, social history of structural racism, religious beliefs, or culture. Third, vaccine intentions fluctuate in response to media reports of relevant current events, but we ascertained intentions at only one time point. Fourth, health policy requires an evidence base from more than one study in one country.

COVID-19 is not the last pandemic. More pandemics are predicted in the future. Also, parents’ resistance to vaccinating their children for communicable diseases will remain a concern. This study reports that vaccine intentions are not short-term isolated misunderstandings that can be readily cleared up by delivering more information to adults during a public-health crisis, but rather are part of a person’s lifelong psychological style of misinterpreting information during stressful uncertain situations. The contribution of our study is the appreciation that this unhelpful pattern of beliefs and behavior is laid down before secondary school age. This timing recommends that national preparation for future pandemics should include age-appropriate preventive education in schools about the epidemiology and biology of viruses, mechanisms of infection, infection-mitigating behaviors, and vaccines. Indeed, Dunedin Study members were asked as 15-year-olds to complete a checklist of “things you want to know more about if you are going to be a parent”; 73% checked “immunisations.”

Such early education can prepare the public to appreciate the need for infection-reducing behaviors such as mask-wearing, social distancing, and vaccination. Today’s Vaccine-Hesitant and Resistant individuals are stuck in an uncertain situation where fast-incoming and complex information about vaccines generates extreme negative emotional reactions (and where pro-vaccination messaging must vie against anti-vaccination messaging that amplifies extreme emotions). Unfortunately, these individuals appear to have diminished capacity to process the information on their own. The results here suggest that, to prepare for future pandemics, education about viruses and vaccines before or during secondary schooling could reduce citizens’ level of uncertainty in a future pandemic, prevent ensuing extreme emotional distress reactions, and provide people with a pre-existing knowledge framework and positive attitudes that enhance receptivity to future health messaging (Ecker et al. 2022). Moreover, many of the factors in the backgrounds of Vaccine-Hesitant and -Resistant Dunedin participants are factors that could be tackled to improve population health in general, such as childhood adversity, low reading levels, mental health, and health knowledge (Galea, 2021).

The newsmedia are reporting recommendations from many quarters about how governments should prepare for future pandemics. Most of these national-strategy recommendations involve medical-technology solutions including more vaccine research-and-development for tests, vaccine delivery, and therapeutics and better-prepared hospitals and monitoring systems, or economic solutions such as a world pandemic fund, more resilient supply chains, and global coordination of vaccine distribution (Adashi & Cohen, 2022; National Academies, 2021; Nuzzon and Gostin, 2022). Technology and money will undoubtedly be important, but a prepared public who understands the need to mask, social distance, and vaccinate will be a valuable asset too.

## Supplementary Material

Supplementary Material

## Figures and Tables

**Table 1.** Dunedin Study cohort members’ 2021 vaccine intentions were matched to archived Study data in order to identify psychological histories of COVID-19 vaccine hesitance and resistance.

Archival measure	Rationale
**Educational attainment and socioeconomic statusn**	Confirm similarity to vaccine-intention groups identified in other surveys in developed nations and lend confidence that this New Zealand cohort can inform health messaging elsewhere.
**Experience with health-care in adulthood**	Test speculation that some people are not vaccinated because their lives do not include familiarity with the health system (Nuffield Council on Bioethics, 2021; Tufecki, 2021).
**Adverse Childhood Experiences (ACEs)**	Test hypothesis that resistant and hesitant groups may have adverse experiences, including abuse or neglect, that underlie a propensity to mistrust that has been present in their lives since childhood.
**Personality in adolescence**	Test hypothesis that resistant and hesitant groups have attitudes, values, motivations and preferences, present since secondary-school age, that increase negativity toward public health messaging through several mechanisms, including tendencies to misinterpret information when under stress, approach messaging with a hostile attribution bias, or dislike messages that seem to limit freedom of personal choice. Personality measures can also reveal whether the vaccine-intention groups differ on pro-vaccination motives such as a desire to care for others, or to keep themselves from harm, or openness to engagement with new information.
**Mental health histories from adolescence to adulthood**	Test hypothesis that the groups differ on longstanding problems such as depression, anxiety, inattention, substance abuse, or thought disorder that can interfere with receipt of vaccine health messaging, or with decision-making, or render people vulnerable to paranoid conspiracy theories.
**Cognitive abilities & reading skill, in childhood, adolescence, and adulthood**	Test hypothesis that messaging for hesitant and resistant groups should be tailored to abilities that influence comprehension and retention of messages.
**Health locus of control in adolescence**	Test hypothesis that teens with external locus of control may become adults who tend to feel helpless or apathetic about making health decisions.
**Comprehension of health knowledge in adulthood**	Test whether health education tailored to pupils’ learning styles might enhance vaccine uptake in future pandemics.

Note: The premise and analysis plan for this project were pre-registered at https://sites.duke.edu/moffittcaspiproiects/files/2021/08/Moffitt_2021a_Covid_vax.pdf

## Data Availability

The Dunedin Study data are available on request by qualified scientists. Requests require a concept paper describing the purpose of data access, ethical approval at the applicant’s institution, and provision for secure data access. We offer secure access on the Duke University and Otago University campuses. All data analysis scripts and results files are available for review.

## Significance Statement

Public-health professionals aspire to tailor pro-vaccination messaging to the backgrounds of vaccine-hesitant or vaccine-resistant people. But who are these people? This 5-decade longitudinal study revealed that vaccine-hesitancy and resistance are not merely misunderstandings by un-informed adults. Intentions emerge from adverse childhood experiences that leave a legacy of mistrust, chronic mental-health problems, lifelong tendencies to have extreme fear and anger emotions and shut down mentally under stress, fatalism about health, non-conformism, and information-processing deficits that, when combined with extreme emotions, can yield decisions that seem inexplicable to health professionals. Vaccine intentions’ roots begin before secondary-school age. Education to prepare pupils to make decisions as adults about infection-reducing behaviors, including vaccinations, should be part of the national preparation strategy for future pandemics.
