# Supplementary material for: Deep-seated psychological histories of COVID-19 vaccine hesitance and resistance
Source: PNAS Nexus. 2022 Mar 24;1(2):pgac034. doi: 10.1093/pnasnexus/pgac034 (PMC9245853; doi:10.1093/pnasnexus/pgac034)
Supplement: pgac034_Supplemental_File [file pgac034_supplemental_file.pdf]

Supplemental Information

Moffitt et al., Deep-seated psychological histories of COVID-19 vaccine hesitance and resistance

1. Study Design and Population 2
2. Vaccine Intention Survey 3
2.1. Reasons why you may not want a vaccine 3
2.2. Reasons why you may want a vaccine 4
2.3. Who do you trust 5
3. Correlates and Predictors of Vaccine Intentions 6
3.1. Educational attainment 6
3.2. Socioeconomic status 6
3.3. Exposure to Adverse Childhood Experieces (ACEs) 6
3.4. Measuring personality: Self reports 7
3.5. Measuring personality: Informant reports 8
3.6. Measuring mental disorders 8
3.7. Childhood IQ 9
3.7. Reading skill 9
3.9. Verbal ability and processing speed 9
3.10. Health knowledge 9
3.11. Health locus of control 10
4. Statistical Analysis 11
Supplementary Table S1 12
Supplementary Table S2 13
Supplementary Table S3 14
Supplementary Table S4 15
Supplementary Table S5 16
Supplementary Figure S1 17
Supplementary Figure S2 18
Supplementary Figure S3 19
Supplementary Figure S4 20
5. References 22

Study Design and Population

Participants are members of the Dunedin Study, a longitudinal investigation of health and behavior in a representative birth cohort. The 1037 participants (91% of eligible births) were all individuals born between April 1972 and March 1973 in Dunedin, New Zealand, who were eligible based on residence in the province and who participated in the first assessment at age 3 years (Poulton et al. 2015). The cohort represents the full range of socioeconomic status (SES) in the general population of New Zealand's South Island and, as adults, matches the New Zealand National Health and Nutrition Survey on key adult health indicators (e.g., body mass index, smoking, and general practitioner visits) and the New Zealand Census of citizens of the same age on educational attainment (Poulton et al. 2015; Richmond-Rakerd et al. 2020). The cohort is primarily white (93%, self-identified), matching South Island demographic characteristics. General assessments were performed at birth as well as ages 3, 5, 7, 9, 11, 13, 15, 18, 21, 26, 32, 38, and, most recently (completed April 2019), at age 45 years, when 938 of the 997 Study members (94.1%) still alive participated. At each assessment, each participant was brought to the research unit for interviews and examinations.

Between March-July 2021, we contacted living Study members (by now N=994). Our analysis is restricted to Study members who were living in New Zealand and Australia (N=942); 88% (N=832) responded to our survey. We conducted an attrition analysis using childhood socioeconomic status (SES) (Elley & Irving, 1976), childhood IQ (Wechsler, 1974), and *p*-factor scores indexing psychopathology from age 18-45 years (Caspi et al. 2020) to determine whether participants in the vaccine intention survey were representative of the original cohort. Responding and non-responding Study members did not differ much on their childhood social class origins ($t(129.7) = 1.43$, $p = .155$), IQ ($t(114.62) = 1.98$, $p = .051$) or history of psychopathology ($t(916) = 1.69$, $p = .091$).

The Dunedin Study was approved by the NZ-HDEC (Health and Disability Ethics Committee). Study members gave informed consent before participating. This study follows the Strengthening the Reporting of Observational Studies in Epidemiology (STROBE) reporting guidelines. The premise and analysis plan for this project were pre-registered at https://sites.duke.edu/moffittcaspiprojects/files/2021/08/Moffitt_2021a_Covid_vax.pdf.

At the time of our survey, only 3 Study members had tested positive for COVID-19. They were living in Europe and are not part of this article. The New Zealand vaccination rollout began in August, after we finished collecting our data in July 2021. As of December 1, 2021, 84% of the eligible New Zealand population between 45-49 years of age has received two doses of the COVID-19 vaccine.

Vaccine Intention Survey

The vaccine intention survey was administered between April-July 2021. Our questions are from the COVID Collaborative (https://www.covidcollaborative.us/; https://covid-19.parc.us.com), Duke University's RADx-UP (https://radx-up.org/learning-resources/cdes/), and the UK COVID-19 Study (https://www.thelancet.com/journals/lanepe/article/PIIS2666-7762(20)30012-0/fulltext). Items ask about intentions, COVID experiences, plus reasons against getting a vaccine, reasons for getting a vaccine, and trusted information sources.

Reasons why you may not want a vaccine.

Study members were provided with a list of reasons about why they may not want a vaccine. For each reason, they were asked whether it applied to them. A principal component analysis of the reasons pointed to four components with eigenvalues greater than 1, accounting for 45.8% of the variance. The items and their standardized component loadings are shown on the next table. The first component reflects Pandemic Doubts; the second component reflects Vaccine Concerns; the third component reflects personal Medical Reasons; the fourth component reflects Personal Circumstances: "It's Not for Me."

Item & Description		Standardized Component Loadings			
		C1	C2	C3	C4
S	Think natural immunity after getting COVID is better than immunity from a vaccination	**0.69**	0.12	-0.05	0.28
R	Would prefer to use alternative medicine to keep safe, such as supplements	**0.68**	0.06	0.16	0.01
G	Don't believe the COVID-19 pandemic is as bad as some people say it is	**0.66**	0.16	-0.08	-0.01
V	Think that the information we get about vaccines from government is not trustworthy	**0.65**	**0.34**	0.11	-0.06
Q	Vaccines go against my religious or personal beliefs	**0.55**	-0.03	0.06	0.05
M	If you still must wear a mask and social distance, what's the point	**0.54**	0.19	0.08	**0.32**
E	Don't think vaccines work very well	**0.52**	0.19	0.19	0.07
C	Not concerned about getting really sick from COVID-19	**0.50**	0.01	-0.15	-0.04
T	Drug companies promote vaccination for financial gain, not for people's health	**0.50**	**0.42**	0.08	0.12
K	Think it was developed too fast, not enough quality control	0.20	**0.73**	-0.04	0.00
D	Concerned about side effects from the vaccine	0.10	**0.73**	0.05	0.03
L	We don't know about the long-term effects of the vaccine	0.04	**0.73**	-0.01	0.03
I	Don't know enough about how well a COVID-19 vaccine works	0.04	**0.69**	0.11	0.11
F	Don't trust that the vaccine will be safe	**0.46**	**0.56**	0.01	-0.02
J	Have a health condition that increases my risk of side effects	0.02	0.09	**0.76**	0.02
A	Allergic to vaccines	0.08	-0.01	**0.76**	0.01
B	Don't like needles or injections	-0.04	-0.09	-0.02	**0.66**
H	Don't want to pay for it	-0.04	0.08	0.00	**0.57**
O	Think that enough other people will be vaccinated to stop the spread of the disease	0.21	0.11	0.01	**0.52**
P	My friends, family or co-workers are against getting vaccinated	**0.36**	0.00	0.13	**0.41**

Reasons why you may want a vaccine.

Study members were provided with a list of reasons about why they may want a vaccine. For each reason, they were asked whether it applied to them. A principal component analysis of the reasons revealed four components with eigenvalues greater than 1, accounting for 57.8% of the variance. The items and their standardized component loadings are shown on the next table. The first component reflects Community Safety; the second component reflects a desire to Return to Normal; the third component reflects Medical Reasons; the fourth component reflects Personal Circumstances: Employment.

Item & Description		Standardized Component Loadings			
		C1	C2	C3	C4
K	Think me getting vaccinated will keep my community safe	**0.88**	0.03	0.04	0.13
J	Think me getting vaccinated will keep my family and friends safe	**0.85**	0.00	0.06	0.19
L	Think getting vaccinated will keep me safe	**0.84**	0.05	0.05	0.13
E	Believe life won't go back to normal until most people get a COVID-19 vaccine	**0.66**	**0.33**	-0.05	-0.10
C	Think the economy depends on everyone getting vaccinated	**0.51**	**0.44**	0.01	0.02
D	Believe COVID-19 can make you really sick	**0.50**	0.20	0.05	0.00
H	After getting vaccinated, I will not have to wear a mask or keep distant from others	-0.03	**0.66**	0.02	0.00
M	Vaccine card will allow me to travel, work, or enter places of business	0.23	**0.62**	0.00	0.10
I	My friends, family, or co-workers say they want to get vaccinated	0.22	**0.54**	0.08	0.32
A	Have a health problem that increases my risk of dying from COVID-19	0.08	-0.03	**0.81**	-0.05
B	Doctor told me to get a COVID-19 vaccine	0.01	0.09	**0.79**	0.10
G	My job places me at high risk of getting COVID-19	0.12	-0.01	-0.01	**0.80**
F	My employer wants me to get vaccinated	0.04	0.22	0.06	**0.77**

Who do you trust?

Study members were provided with a list of sources of advice and asked whether they trusted those sources or not. A principal component analysis revealed three components with eigenvalues greater than 1, accounting for 50.3% of the variance. The items and their standardized component loadings are shown on the next table. The first component reflects Trust in Institutions; the second component reflects Trust in Friends & Family; the third component reflects Trust in Influencers.

Item & Description		Standardized Component Loadings C1	C2	C3
K	The government	**0.78**	0.03	0.11
J	Scientists	**0.77**	0.03	0.00
A	Your doctor or health care provider	**0.65**	0.14	-0.10
F	News on the radio, TV, online, and newspapers	**0.48**	0.16	**0.42**
I	Drug companies	**0.40**	0.14	**0.36**
D	Members of your family	0.11	**0.85**	-0.04
C	Your close friends	0.11	**0.85**	0.07
E	People you work with or other people you know	0.15	**0.62**	0.29
B	Your faith leader, minister, priest, pastor	0.01	**0.31**	0.13
G	Celebrities you admire	0.00	0.07	**0.72**
H	Your contacts on social media	0.02	0.18	**0.70**

Correlates and predictors of vaccine intentions

All measures have been described in previous reports from the Dunedin Study. Here we provide a summary of the measures and references for further details.

Educational attainment.

We measured educational attainment as the highest degree completed by a Dunedin Study member through the time of the age-45 assessment. For the 1972-73 birth cohort we studied, compulsory education ended at age 15 years, at which point students could elect to sit for a School Leaving Certificate exam. 14% of our sample obtained no educational credential. 15% obtained the School Leaving Certificate but did not progress further. 41% completed 6th form or Bursary Certificates (roughly equivalent to a full high school diploma in the United States). 30% completed a university degree.

Socioeconomic status.

Study members' socioeconomic status at age 45 was measured according to the New Zealand Socio-Economic Index 2006 (NZSEI-06), a six-group occupation-based measure of socioeconomic status (Milne et al. 2013). Examples of occupations in the 6 groups include medical practitioner (group 6), engineering professional (group 5), data-base administrator (group 4), personal assistant (group 3), office cashier (group 2), and fish filleter (group 1).

Exposure to Adverse Childhood Experiences (ACEs).

As previously described (Reuben et al. 2016), we assessed 10 categories of ACEs introduced by the CDC–Kaiser Permanente Adverse Childhood Experiences Study (Felitti et al. 1998): 5 types of child harm (physical abuse, emotional abuse, physical neglect, emotional neglect, and sexual abuse) and 5 types of household dysfunction (incarceration of a family member, household substance abuse, household mental illness, loss of a parent, and household partner violence). Reported ACEs were derived from structured interviews conducted with Dunedin Study members at age 38, during which Study members were asked about childhood experiences. The number of these adversities experienced yielded a cumulative ACEs score, range: 0-9.

Measuring Personality: Multidimensional Personality Questionnaire (MPQ).

At age 18 years, Study Members completed a modified 177-item version of the Multidimensional Personality Questionnaire (MPQ; Patrick et al. 2002) adapted for use in New Zealand (Krueger et al., 1996; Caspi et al., 1997; Caspi 2000). As shown on the next table, the personality scales composing the MPQ can be viewed at the higher-order level as defining 3 distinct superfactors (Positive Emotionality, Negative Emotionality, and Constraint) and at the lower-order level as defining 10 more basic aspects of personality variation (an 11th lower-order scale, Absorption, was not included in this modification of the MPQ). Individuals scoring high on the dimension of Positive emotionality have a lower threshold for the experience of positive emotions and for positive engagement in their social and work environments, and they tend to view life as being essentially a pleasurable experience. The Positive Emotionality scale is a combination of scores from the lower-order MPQ scales of Well-being, Social Potency, Achievement, and Social Closeness. Individuals scoring high on the dimension of Negative Emotionality have a low general threshold for the experience of negative emotions such as anxiety and anger, and they tend to break down under stress. The Negative Emotionality scale is a combination of scores from the lower-order MPQ scales of Stress Reactivity, Alienation, and Aggression. Individuals scoring high on the dimension of Constraint tend to endorse conventional social norms, avoid thrills, and act in a cautious and restrained manner. The Constraint scale is a combination of scores from the lower-order MPQ scales of Self-control, Harm Avoidance, and Traditionalism. Scores were standardized to a mean of 0 [SD, 1].

***Multidimensional Personality Questionnaire (MPQ) scale descriptions*.**

MPQ Scale	Description of high scorer
**Positive Emotionality**	
Well-Being	Has a happy, cheerful disposition; feels good about self, sees a bright future.
Social Potency	Is forceful and decisive; enjoys influencing others; fond of leadership roles.
Achievement	Works hard; enjoys demanding projects and working long hours.
Social Closeness	Is sociable, likes people, and turns to others for comfort.
**Negative Emotionality**	
Stress Reactivity	Is nervous, vulnerable, sensitive, prone to worry, shuts down under stress.
Alienation	Feels mistreated, victimized, betrayed, and the target of false rumors.
Aggression	Willing to hurt others for own advantage; will frighten and cause discomfort
**Constraint**	
Self-Control	Is reflective, cautious, careful, rational, planful.
Harm Avoidance	Avoids excitement and danger; prefers safe activities even if tedious.
Traditionalism	Socially conservative, conformist, favors social norms and rules.

Measuring Personality: Informant Reports of the Big Five Personality Traits.

At ages 26, 32, 38, and 45 years, we also measured the personalities of Dunedin participants through reports by co-informants (mostly best friends, partners, or other family members), as previously described (Wertz et al., 2021). At each age, informants were mailed questionnaires asking them to describe the Study member using a brief, 25-item version of the Big 5 Inventory (Benet-Martínez and John, 1998) measuring the personality traits of Openness to experience ("Original, comes up with new ideas"), Conscientiousness ("Works until a thing is done"), Extraversion ("Outgoing, likes people"), Agreeableness ("Kind and considerate"), and Neuroticism ("Gets nervous easily"). We age-standardized and then averaged each of the personality traits across the 4 phases to yield a profile of Dunedin participants' adult personalities.

Measuring Mental Disorders: Externalizing, Internalizing, and Thought Disorders.
Mental disorders are disturbances in thought, behavior, and emotion that interfere with or limit social, family, educational, or work activities. In the Dunedin Study, these were identified according to the criteria of the Diagnostic and Statistical Manual of Mental Disorders (DSM) as previously described (Caspi et al. 2020). Psychiatric interviews were carried out by health professionals at ages 18, 21, 26, 32, 38, and 45 with the Diagnostic Interview Schedule (Robins 1981, 1995). At ages 18 and 21, diagnoses were made according to DSM-III-R (APA, 1987); at ages 26, 32, and 38, according to DSM-IV (APA 2000); at age 45 according to DSM-V (APA 2013). Using Confirmatory Factor Analysis (CFA), we estimated three latent factors, each of which influences a subset of psychiatric symptoms: Externalizing Disorders (with loadings from ADHD, conduct disorder, alcohol, cannabis, tobacco, and other drug dependence), Internalizing Disorders (with loadings from depression, generalized anxiety disorder, fears/phobias, PTSD, eating disorders) and Thought Disorders (with loadings from obsessive-compulstive disorder, mania, and positive and negative schizophrenia symptoms). The model fit the data well: χ^2(2465, N=1,000) = 4082.230, CFI = .933, TLI = .929, RMSEA = .026, 90% confidence interval (CI) = [.024, .027], confirming that three correlated factors (i.e., Internalizing, Externalizing, and Thought Disorder) explain well the structure of the disorder symptoms examined across 27 years from late adolescence to midlife.

Childhood IQ.

The Wechsler Intelligence Scale for Children-Revised (WISC-R; Wechsler, 1974) was administered to the Study members at ages 7, 9, and 11 years. IQ scores for the three ages were averaged and standardized to a mean of 100 [SD, 15]. For readers interested in cognitive epidemiology, references are at the end of this supplement (Deary, 2010; Gottfredson, 2004; Lubinsky & Humphreys, 1997).

Reading skill.

Reading skill was individually assessed at age 18 years using the Burt Word Reading Test (Scottish Council for Research in Education, 1976), a word recognition reading test consisting of 110 selected words in isolation that resembles the American Wide Range Achievement Test of reading. Scores were standardized to a mean of 100 [SD,15].

Verbal ability and Processing Speed.

Cognitive ability was measured at age 45 using the Wechsler Adult Intelligence Scale IV (WAIS-IV) full-scale IQ; range, 40-160, standardized to a mean of 100 [SD, 15]) (Wechsler, 2008). We report scores for the Verbal Comprehension Index, which measures the ability to use and think with spoken language, and the Processing Speed Index, which measures the ability to make quick and accurate decisions efficiently.

Health Knowledge.

Study members' practical health knowledge at age 45 was indexed by two scales (Richmond-Rakerd et al., 2020):

Multiple-choice assessment. Participants were administered a six-item multiple-choice assessment of their understanding of different health principles, including those related to medical knowledge, prevention, aging, physical disease, sun exposure, and sleep. The number of correct responses was summed to create a scale (range=0-6).

Open-ended interview. Participants were interviewed about their understanding of different health principles, with an open-ended response format: “What are some of the reasons you should know your family history of illness?”; “If you are sick and the doctor gives you an antibiotic, what are some of the reasons why you should finish all the pills?”; “What are some of the reasons it is important to get your blood pressure checked?”; “What are some of the reasons you should wear sunglasses when out on a sunny day?” “What are some of the reasons you should get regular sleep?”; and “What are some of the reasons people tend to gain weight as

they get older?” Using standardized scoring procedures, four trained raters (two raters per interview) coded responses on a scale from 0 to 2, with 0 indicating no understanding of the health principle, 1 indicating moderate understanding, and 2 indicating good understanding (interrater reliability=0.94). For instance, in response to the question “What are some of the reasons you should wear sunglasses when out on a sunny day?”, the following responses were coded as 0, 1, and 2, respectively: “To prevent squinting,” “To prevent eye damage,” and “To protect your eyes from UV rays.” Scale scores were computed by summing across the items and then averaging across raters (range=1-12). The multiple-choice and open-ended scales were correlated ($r=0.39$, $p<.0001$). The Practical Health Knowledge measure was computed by standardizing ($M=0$, $SD=1$) and averaging the multiple-choice and open-ended scales.

Health Locus of Control.

When the Study members were 13 and 15 years old, they were administered the Multidimensional Health Locus of Control scales (Stanton et al., 1995), which measure the extent to which people believe they have the power to control factors that affect their health versus that health outcomes are due to external factors or chance. The measure is scored such that higher scores reflect more external factors or chance.

Statistical analysis

We used Ordinary Least Squares regression to compare the Vaccine Resistant and Vaccine Hesitant to the Vaccine Willing. Models were run in MPlus (v8.5, Muthen & Muthen, 1998-2020) using full information maximum likelihood (FIML) and bootstrapped (N = 1000) standard errors. All tests controlled for sex. All regression coefficients represent sex-adjusted mean differences in standardized units and can be compared across variables. Statistical significance was evaluated using an alpha level of 0.05. No adjustment to the alpha level was made because we tested prespecified hypotheses, used previously published predictor measures, analyzed outcomes that were correlated with each other, reported results for all tests, and did not test a universal null hypothesis (Armstrong, 2014). Analyses were checked by an independent data analyst for reproducibility creating new code from the manuscript and applying it to a copy of the original data. High retention means missing data for all predictor variables were not a problem. We assessed >90% of the cohort at 12 of 13 Study phases. Attrition is not cumulative or systematic; different individuals are missing at any given phase; data are missing at random allowing us to apply FIML.

Table S1. Educational attainment and socioeconomic status among the Vaccine-Willing, Vaccine-Hesitant and Vaccine-Resistant groups.

	Sex-adjusted group means						Standardized, sex-adjusted comparisons between groups					
	Vaccine Willing		Vaccine Hesitant		Vaccine Resistant		Willing vs Hesitant			Willing vs Resistant		
	Mean	95% CI	Mean	95% CI	Mean	95% CI	B	95% CI	p	B	95% CI	p
Educational Attainment	2.01	(1.92, 2.08)	1.53	(1.33, 1.72)	1.39	(1.19, 1.57)	-.50	(-.70, -.28)	**<.001**	-.62	(-.82, -.42)	**<.001**
SES at age 45 years	3.91	(3.80, 4.01)	3.10	(2.85, 3.36)	3.09	(2.82, 3.35)	-.57	(-.75, -.38)	**<.001**	-.56	(-.76, -.37)	**<.001**

Table S2. Reasons against vaccination, reasons for vaccination, and sources of trust during the COVID-19 pandemic.

	Sex-adjusted group means						Standardized, sex-adjusted comparisons between groups					
	Vaccine Willing		Vaccine Hesitant		Vaccine Resistant		Willing vs Hesitant			Willing vs Resistant		
	Mean[a]	95% CI	Mean[a]	95% CI	Mean[a]	95% CI	B	95% CI	p	B	95% CI	p
Reasons for not wanting COVID vaccine												
Vaccine Concerns	.41	(.38,.43)	.75	(.69,.80)	.84	(.80, .88)	.98	(.80, 1.14)	**<.001**	1.27	(1.13, 1.41)	**<.001**
Pandemic Doubts	.06	(.05,.07)	.19	(.15,.23)	.40	(.35, .45)	.69	(.47, .91)	**<.001**	1.78	(1.51, 2.05)	**<.001**
Medical Reasons	.05	(.04,.06)	.11	(.06,.16)	.10	(.06, .14)	.34	(.08, .61)	**.018**	.29	(.07, .53)	**.015**
Personal Circumstances	.09	(.08,.10)	.12	(.08,.16)	.14	(.11, .18)	.19	(-.03, .43)	.106	.32	(.11, .58)	**.007**
Reasons for wanting COVID vaccine												
Community Safety	.81	(.79,.83)	.48	(.41,.55)	.22	(.17, .28)	-.97	(-1.18, -.76)	**<.001**	-1.74	(-1.90, -1.57)	**<.001**
Return to Normal	.39	(.37,.42)	.31	(.25,.36)	.25	(.20, .29)	-.30	(-.51, -.11)	**.002**	-.52	(-.71, -.35)	**<.001**
Medical Reasons	.11	(.10,.14)	.08	(.04,.12)	.03	(.01, .06)	-.14	(-.33, .06)	.165	-.34	(-.47, -.22)	**<.001**
Personal Circumstances	.25	(.22,.27)	.15	(.10,.20)	.15	(.10, .21)	-.28	(-.46, -.11)	**.002**	-.29	(-.46, -.10)	**.002**
Trust in sources of COVID advice												
Institutions	.52	(.51,.54)	.27	(.23,.31)	.19	(.15, .23)	-.91	(-1.07, -.75)	**<.001**	-1.19	(-1.37, -1.02)	**<.001**
Friends & Family	.22	(.20,.25)	.19	(.14,.24)	.12	(.07, .16)	-.12	(-.32, .09)	.263	-.38	(-.56, -.21)	**<.001**
Influencers	.02	(.01,.03)	.01	(.00,.03)	.01	(.00, .03)	-.09	(-.23, .09)	.292	-.05	(-.22, .13)	.600

[a] Scores represent the proportion of items endorsed.

Table S3. Childhood experiences, adolescent personality profiles, and mental-health histories of the Vaccine-Willing, Vaccine-Hesitant and Vaccine-Resistant groups.

	Sex-adjusted group means						Standardized, sex-adjusted comparisons between groups					
	Vaccine Willing		Vaccine Hesitant		Vaccine Resistant		Willing vs Hesitant			Willing vs Resistant		
	Mean	95% CI	Mean	95% CI	Mean	95% CI	B	95% CI	p	B	95% CI	p
Adverse Childhood Experiences (ACEs)												
	1.46	(1.32, 1.59)	1.86	(1.50, 2.24)	2.25	(1.84, 2.64)	.21	(-.01, .43)	.052	.44	(.19, .66)	**<.001**
MPQ Personality, age 18y												
Positive Emotionality	.05	(-.04, .12)	-.15	(-.35, .04)	-.12	(-.30, .07)	-.19	(-.41, .04)	.087	-.17	(-.36, .03)	.091
Negative Emotionality	-.09	(-.17, -.01)	.21	(.02, .40)	.33	(.15, .52)	.32	(.11, .53)	**.002**	.42	(.21, .61)	**<.001**
Constraint	.04	(-.03, .11)	-.03	(-.21, .16)	-.22	(-.40, -.03)	-.11	(-.31, .09)	.256	-.25	(-.45, -.05)	**.013**
Well-Being	.04	(-.03, .12)	-.13	(-.34, .08)	-.12	(-.34, .08)	-.16	(-.38, .06)	.147	-.17	(-.40, .05)	.144
Social Potency	.02	(-.07, .09)	-.06	(-.24, .13)	-.03	(-.22, .16)	-.07	(-.26, .14)	.516	-.05	(-.26, .17)	.621
Achievement	.02	(-.06, .10)	-.12	(-.28, .04)	-.01	(-.20, .16)	-.13	(-.30, .05)	.170	-.04	(-.23, .17)	.726
Social Closeness	.04	(-.04, .11)	-.05	(-.25, .15)	-.15	(-.33, .02)	-.11	(-.33, .11)	.313	-.18	(-.38, .02)	.072
Stress Reactity	-.05	(-.13, .02)	.16	(-.02, .35)	.15	(-.02, .33)	.19	(-.01, .40)	.078	.21	(.02, .40)	**.034**
Alienation	-.07	(-.15, .01)	.22	(.01, .43)	.22	(.04, .41)	.31	(.09, .53)	**.005**	.29	(.09, .49)	**.006**
Aggression	-.08	(-.14, -.01)	.09	(-.08, .26)	.36	(.19, .54)	.22	(.02, .41)	**.022**	.43	(.25, .61)	**<.001**
Self-Control	.04	(-.04, .12)	-.17	(-.36, .02)	-.08	(-.25, .08)	-.24	(-.45, -.02)	**.023**	-.12	(-.30, .07)	.184
Harm Avoidance	.00	(-.07, .07)	.10	(-.05, .25)	-.10	(-.30, .10)	.05	(-.11, .22)	.534	-.09	(-.30, .12)	.403
Traditionalism	.06	(-.02, .13)	.02	(-.17, .22)	-.34	(-.55, -.14)	-.05	(-.26, .16)	.647	-.40	(-.62, -.17)	**.001**
Mental Health Histories (ages 18-45y)												
Externalizing	97.9	(96.9, 99.0)	104.1	(100. 8, 107.1)	108.1	(105.0, 111.2)	.44	(.20, .65)	**<.001**	.67	(.46, .89)	**<.001**
Internalizing	98.7	(97.6, 99.8)	104.3	(101.1, 107.4)	103.3	(10.7, 105. 9)	.34	(.13, .57)	**.002**	.31	(.14, .50)	**.001**
Thought Disorders	98.3	(97.2, 99.3)	104.8	(101.4, 108.0)	105.4	(102.6, 108.4)	.43	(.19, .66)	**<.001**	.48	(.28, .68)	**<.001**

Table S4. Cognitive characteristics of the Vaccine-Willing, Vaccine-Hesitant and Vaccine-Resistant groups.

	Sex-adjusted group means						Standardized, sex-adjusted comparisons between groups					
	Vaccine Willing		Vaccine Hesitant		Vaccine Resistant		Willing vs Hesitant			Willing vs Resistant		
	Mean	95% CI	Mean	95% CI	Mean	95% CI	B	95% CI	p	B	95% CI	p
Childhood IQ	101.4	(100.2, 102.5)	94.8	(91.9, 97.5)	97.0	(94.4, 99.5)	-.43	(-.62, -.22)	**<.001**	-.29	(-.48, -.10)	**.002**
Reading, 18y	101.0	(99.8, 102.1)	96.2	(93.0, 99.3)	97.8	(95.4, 100.2)	-.34	(-.56, -.12)	**.002**	-.21	(-.39, -.03)	**.022**
Verbal Comprehension, 45y	101.7	(100.6, 102.9)	92.4	(89.8, 94.8)	97.1	(94.7, 99.6)	-.62	(-.80, -.43)	**<.001**	-.31	(-.48, -.12)	**.001**
Processing Speed, 45y	100.9	(99.8, 102.0)	96.8	(94.0, 99.6)	97.8	(95.3, 100.5)	-.31	(-.51, -.11)	**.004**	-.20	(-.39, .00)	**.050**
Health Knowledge, 45y	101.7	(100.5, 102.8)	94.5	(91.6, 97.4)	95.0	(92.4, 97.6)	-.50	(-.70, -.29)	**<.001**	-.45	(-.62, -.25)	**<.001**
Health Locus of Control, 13-15y	-.04	(-.12, .03)	.07	(-.14, .29)	.20	(-.01, .39)	.12	(-.11, .35)	.324	.24	(.03, .46)	**.027**

Table S5. Childhood experiences, adolescent personality profiles, mental-health histories and cognitive characteristics of the Vaccine-Willing, Vaccine-Hesitant and Vaccine-Resistant groups. The table presents the results of the main associations (shown in Tables S3 and S4) before (Panel A) and after (Panel B) controlling for the socioeconomic origins (SES) of the Study participants. The socioeconomic status of cohort members' families was measured using a six-point scale derived from the New Zealand Census (Elley & Irving, 1976). The deep-seated psychological histories associated with vaccine hesitance and resistance were not simply attributable to the socioeconomic background in which hesitant and resistant individuals grew up. When we controlled for the participants' socioeconomic background, we found that childhood adversity, tendencies toward extreme negative emotions and shutting down mentally under stress, mental health histories, and cognitive difficulties continued to distinguish Vaccine-Hesitant and Vaccine-Resistant groups.

	A. Standardized comparisons between groups: Adjusted for sex						B. Standardized comparisons between groups: Adjusted for sex and childhood SES					
	Willing vs Hesitant			Willing vs Resistant			Willing vs Hesitant			Willing vs Resistant		
	B	95% CI	p	B	95% CI	p	B	95% CI	p	B	95% CI	p
Adverse Childhood Experiences (ACEs)												
	.21	(-.01, .43)	.052	.44	(.19, .66)	**<.001**	.15	(-.07, .36)	.176	.38	(.16, .61)	**.001**
MPQ Personality, age 18y												
Positive Emotionality	-.19	(-.41, .04)	.087	-.17	(-.36, .03)	.091	-.16	(-.38, .06)	.142	-.15	(-.34, .06)	.142
Negative Emotionality	.32	(.11, .53)	**.002**	.42	(.21, .61)	**<.001**	.28	(.06, .48)	**.008**	.38	(.19, .58)	**<.001**
Constraint	-.11	(-.31, .09)	.256	-.25	(-.45, -.05)	**.013**	-.15	(-.34, .05)	.137	-.28	(-.48, -.08)	**.005**
Mental Health Histories (ages 18-45y)												
Externalizing	.44	(.20, .65)	**<.001**	.67	(.46, .89)	**<.001**	.41	(.18, .62)	**<.001**	.65	(.44, .86)	**<.001**
Internalizing	.34	(.13, .57)	**.002**	.31	(.14, .50)	**.001**	.32	(.09, .54)	**.005**	.29	(.11, .48)	**.002**
Thought Disorders	.43	(.19, .66)	**<.001**	.48	(.28, .68)	**<.001**	.39	(.16, .62)	**.001**	.45	(.26, .64)	**<.001**
Childhood IQ	-.43	(-.62, -.22)	**<.001**	-.29	(-.48, -.10)	**.002**	-.30	(-.49, -.10)	**.003**	-.19	(-.37, -.01)	**.032**
Reading, 18y	-.34	(-.56, -.12)	**.002**	-.21	(-.39, -.03)	**.022**	-.24	(-.46, -.02)	**.025**	-.12	(-.30, .05)	.156
Verbal Comprehension, 45y	-.62	(-.80, -.43)	**<.001**	-.31	(-.48, -.12)	**.001**	-.50	(-.68, -.33)	**<.001**	-.21	(-.39, -.04)	**.018**
Processing Speed, 45y	-.31	(-.51, -.11)	**.004**	-.20	(-.39, .00)	**.050**	-.26	(-.46, -.06)	**.011**	-.16	(-.36, .04)	.107
Health Knowledge, 45y	-.50	(-.70, -.29)	**<.001**	-.45	(-.62, -.25)	**<.001**	-.42	(-.61, -.21)	**<.001**	-.38	(-.55, -.19)	**<.001**
Health Locus of Control, 13-15y	.12	(-.11, .35)	.324	.24	(.03, .46)	**.027**	.04	(-.17, .28)	.698	.20	(-.01, .40)	.063

Figure S1. Sex-adjusted Big 5 personality traits, as reported by informants who knew the Study Members well. The Vaccine-Resistant and Vaccine-Hesitant groups scored lower than the Vaccine-Willing group on Conscientiousness (B=-.33 [-0.55, -0.11], P = 0.004 and B=-.32 [-0.54, -0.12], P = 0.002, respectively) and Agreeableness (B=-.56 [-0.76, -0.35], P < 0.001 and B=-.42 [-0.62, -0.20], P < 0.001, respectively), and higher on Neuroticism (B=.33 [0.14, 0.52], P = 0.001 and B=.24 [0.04, 0.44], P = 0.019, respectively). They did not differ on Openness to Experience (B=.01 [-0.19, 0.20], P = 0.951 and B=-.13 [-0.34, 0.10], P = 0.231, respectively) or Extraversion (B=-.12 [-0.33, 0.07], P = 0.227 and B=-.01 [-0.21, 0.19], P = 0.942, respectively).

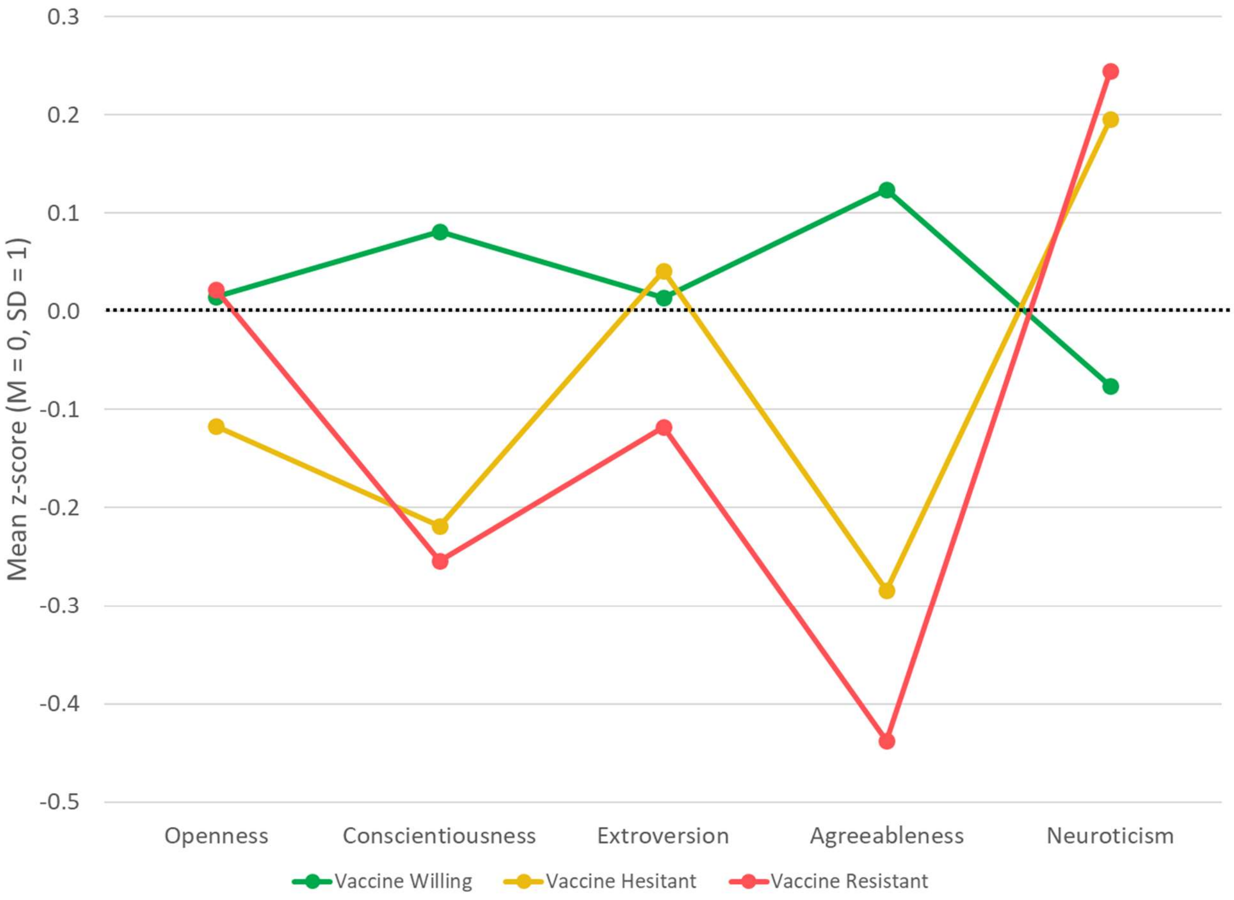

Figure S2. Correlations between reasons for not wanting to get vaccinated and the prospective predictors of vaccine intentions. A principal component analysis identified four reasons for not wanting to get vaccinated: (a) vaccine concerns, (b) pandemic doubts, (c) medical reasons, and (d) personal circumstances. The forest plot show sex-adjusted correlations with 95% confidence intervals.

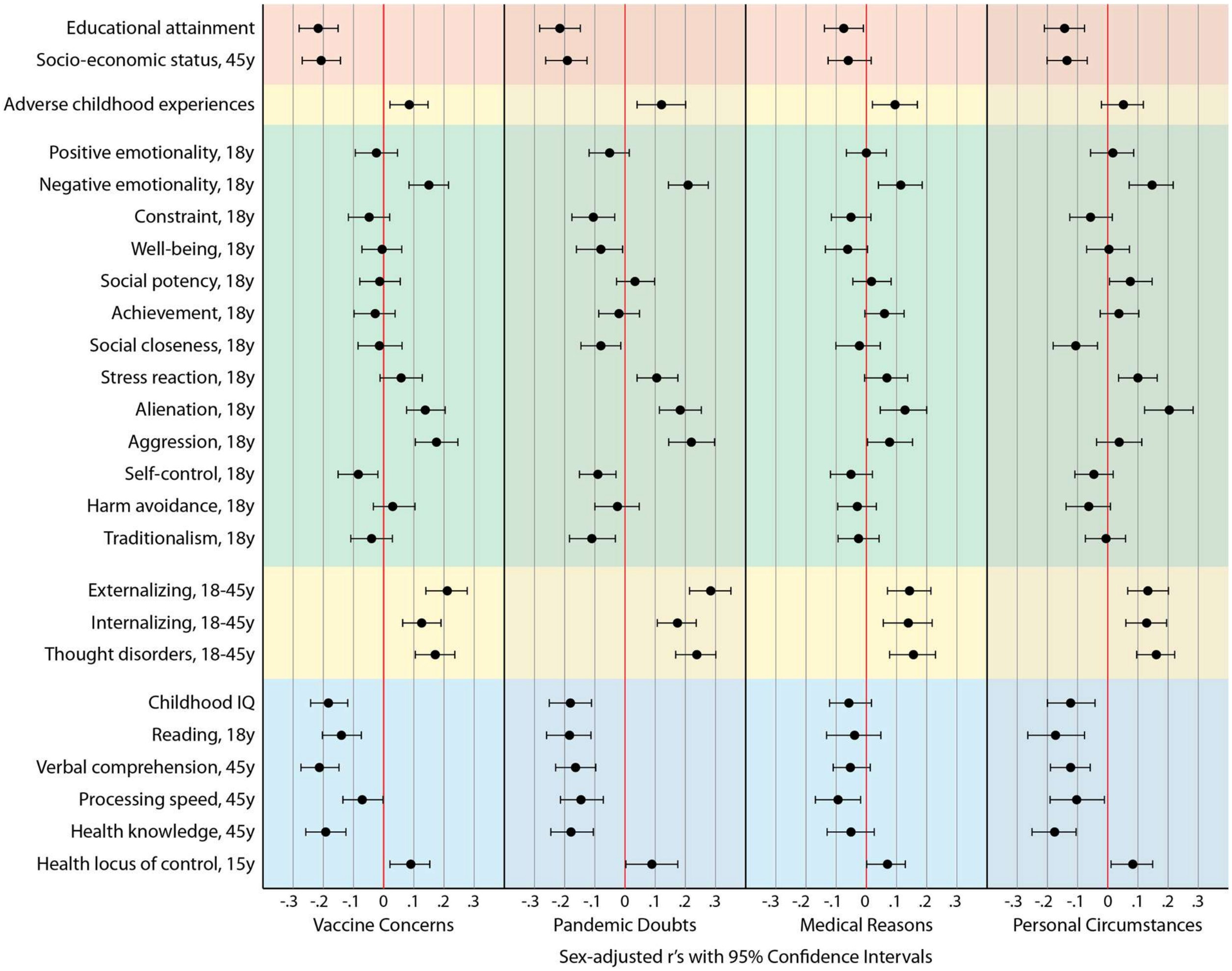

Figure S3. The association between IQ scores and vaccine intentions was the same across low, medium, and high social classes. Note: The group differences in the overall means appear to differ from the group differences among the within-SES group means because the Ns of the 9 SES x Vaccine Intention groups are not balanced (e.g., the Vaccine-Willing Group has 17% low-SES people, while the Vaccine-Hesitant and Vaccine-Resistant have 26% and 28% low-SES people, respectively).

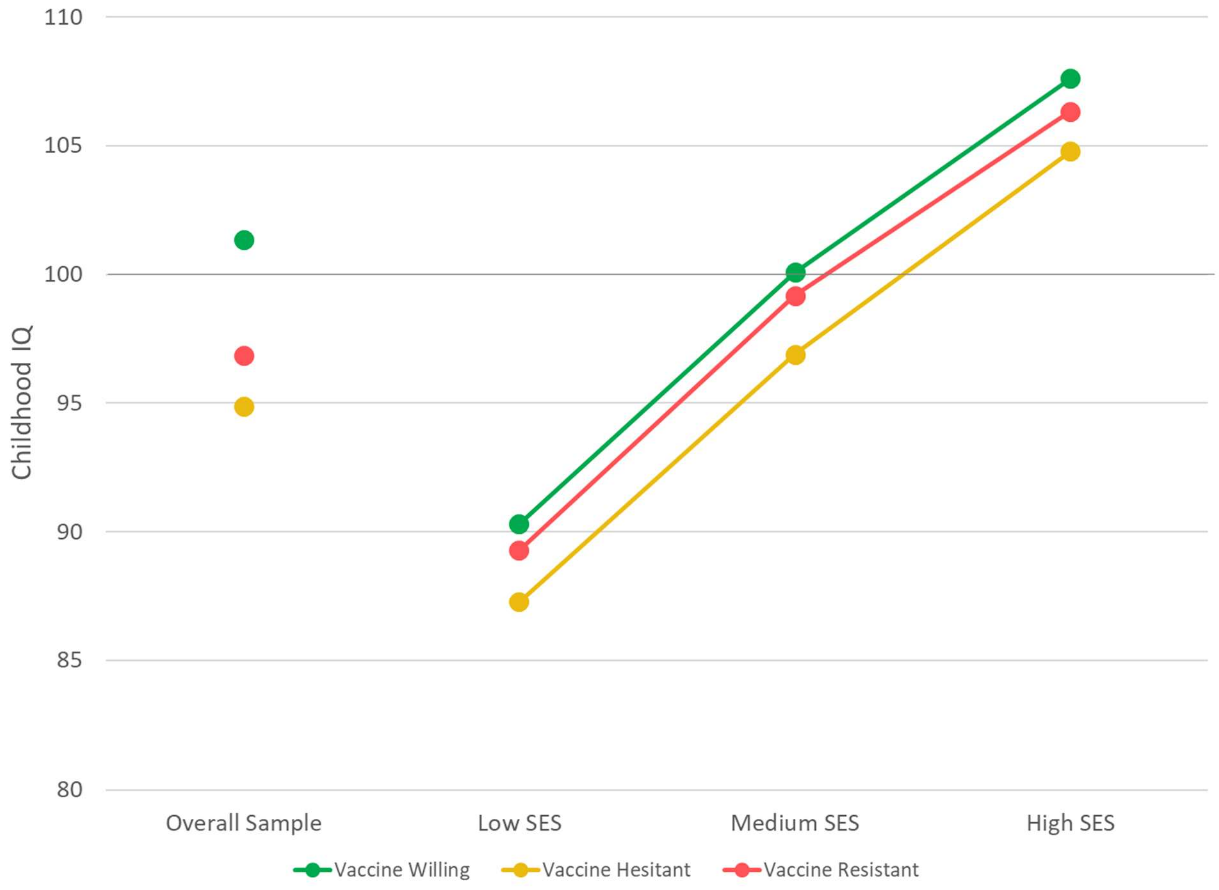

Figure S4. We singled out college-educated Vaccine-Resistant individuals (n=15; only 13% of Resistors) to check if they differed from the less-educated majority of Vaccine-Resistant individuals on key psychological predictors. (As 80% of educated resistors were women, we present sex-adjusted means.) The figure shows college-educated Resistors scored less extremely than the less-educated Resistors. This means that our findings slightly under-estimate associations between predictors and vaccine resistance for the majority of Resistors (i.e., those who have less than a college education). The Figure shows the following key psychological predictors: (**A**) number of Adverse Childhood Experiences (ACEs), (**B**) mean MPQ negative emotionality at age 18 years, (**C**) mean mental health symptom histories from age 18 to 45 years, (**D**) childhood IQ, (**E**) health knowledge at age 45 years, and (**F**) health locus of control at ages 13-15 among the Vaccine-Willing (green), Vaccine-Resistant (red), Low-Education Vaccine-Resistant (coral), and High-Education Vaccine-Resistant (pink). Means are sex-adjusted. Error bars represent 95% Confidence Intervals, to visualize if the confidence intervals between groups overlap.

S4A. Adverse childhood experiences

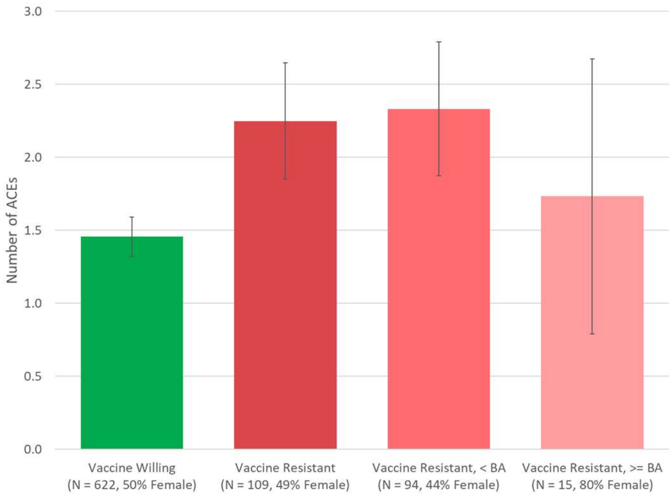

S4B. Adolescent negative emotionality

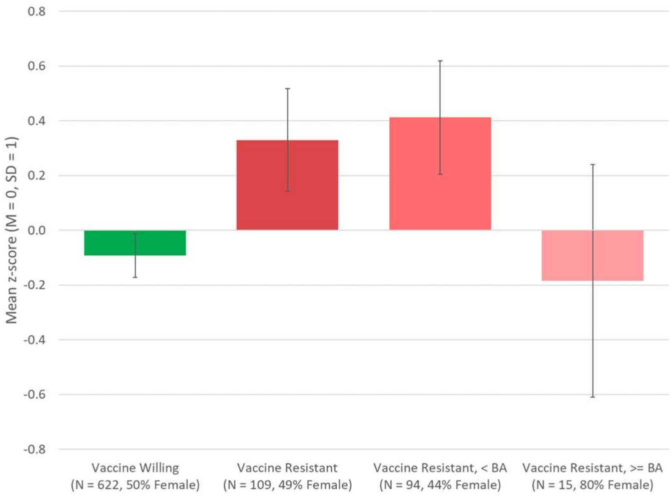

Figure S4, continued.

S4C. Mental health histories, adolescence to adulthood

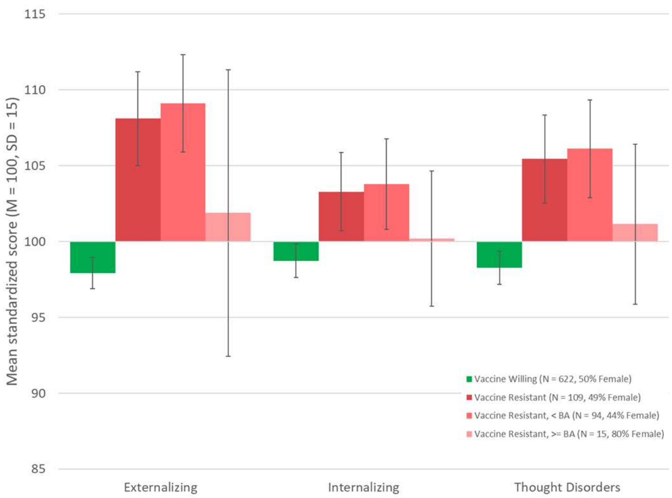

S4D. Childhood IQ

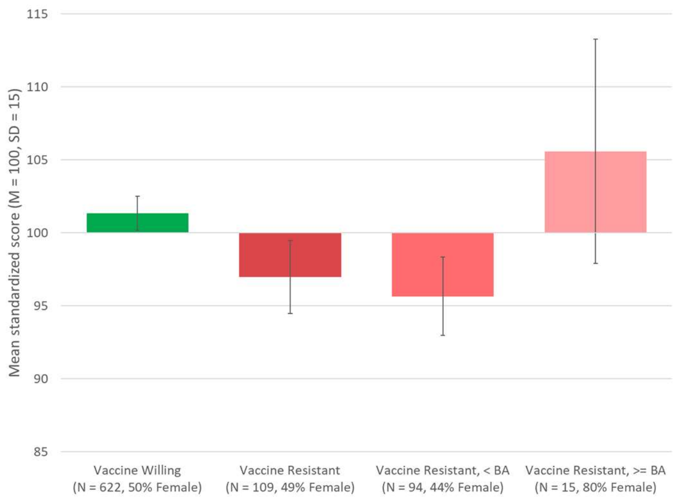

S4E. Health knowledge at age 45 years

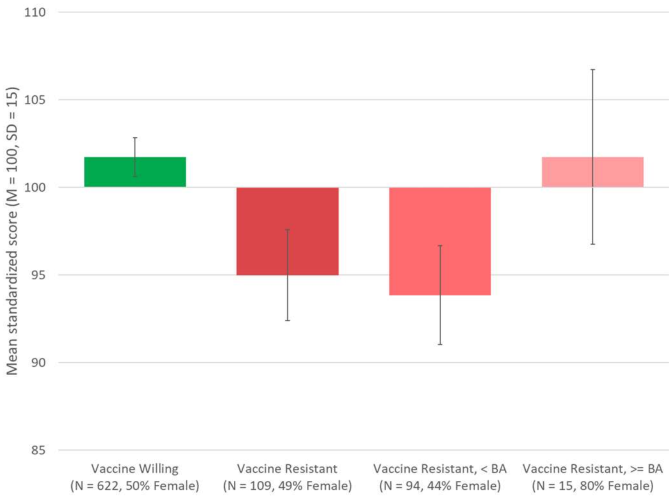

S4F. External health locus of control at ages 13-15

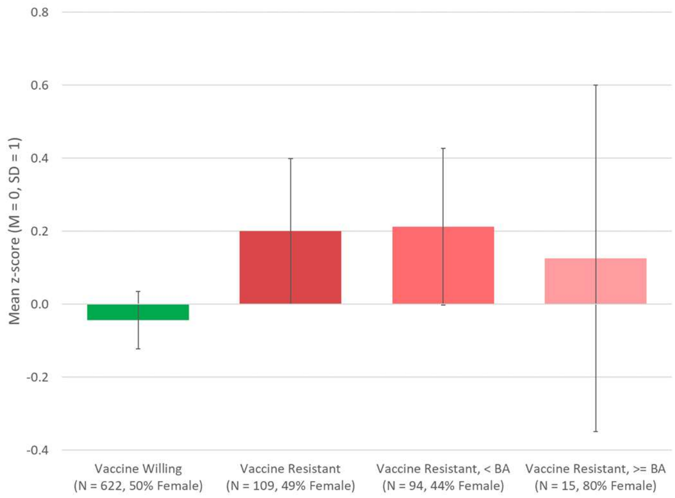

References

APA. (1987). *Diagnostic and Statistical Manual of Mental Disorders*. Revised. Washington, DC: American Psychiatric Association.

APA. (2000). *Diagnostic and Statistical Manual of Mental Disorders (4th Ed., Text Rev.)*. Washington, DC: American Psychiatric Association.

APA. (2013). *Diagnostic and Statistical Manual of Mental Disorders (5th Ed.)*. Washington, DC: American Psychiatric Association.

Armstrong, RA. (2014). *When to use the Bonferroni correction. Ophthalmic and Physiological Optics, 34, 502–508.*

Benet-Martínez, V., John, O.P. (1998). Los cinco grandes across cultures and ethnic groups: multitrait multimethod analyses of the Big five in Spanish and English. Journal of Personality and Social Psychology, 75, 729–750.

Caspi, A. (2000). The child is father of the man: Personality continuities from childhood to adulthood. *Journal of Personality and Social Psychology, 78*, 158–172.

Caspi A. Begg, D. Dickson N, Harrington H-L, Langley J, Moffitt TE, Silva PA. (1997). Personality differences predict health-risk behaviors in adulthood: evidence from a longitudinal study. *Journal of Personality and Social Psychology,* 73, 1052- 1063.

Caspi A, Houts RM, Ambler A, Danese A, Elliott ML, Hariri A, Harrington HL, Hogan S, Poulton R, Ramrakha S, Rasmussen LJH, Reuben A, Richmond-Rakerd L, Sugden K, Wertz J, Williams BS, Moffitt TE. (2020). Longitudinal assessment of mental health disorders and comorbidities across 4 decades among participants in the Dunedin birth cohort study. JAMA Network Open, 4:e203221.

Caspi A, Houts RM, Belsky DW, et al. (2014). The p Factor: One General Psychopathology Factor in the Structure of Psychiatric Disorders? *Clinical Psychological Science,* 2, 119-137.

Caspi A, Moffitt TE. (2018). All for one and one for all: Mental disorders in one dimension. *American Journal of Psychiatry,* 175, 831-844.

Deary, I. J. (2010). Cognitive epidemiology: Its rise, its current issues, and its challenges. *Personality and Individual Differences*, 49, 337-343.

Elley WB, Irving JC. (1976). Revised socio-economic index for New Zealand. New Zealand Journal of Educational Studies,11, 25-36.

Felitti V.J. Anda R.F. Nordenberg D. Williamson D.F. Spitz A.M. Edwards V. et al. (1998). Relationship of childhood abuse and household dysfunction to many of the leading causes of death in adults. The Adverse Childhood Experiences (ACE) Study. *American Journal of Preventice Medicine,* 14, 245-258.

Heatherton TF, Kozlowski LT, Frecker RC, Fagerstrom K-OO. (1991). The Fagerström Test for Nicotine Dependence: A revision of the Fagerstrom Tolerance Questionnaire. *British Journal of Addiction,* 86, 1119-1127.

Gottfredson, L. S. (2004). Intelligence: Is it the epidemiologists' elusive "fundamental cause" of social class inequalities in health. *Journal of Personality and Social Psychology*, 86, 174-199.

Krueger RF, Caspi A, Moffitt TE, Silva PA, McGee R. (1996). Personality traits are differentially linked to mental disorders: a multitrait-multidiagnosis study of an adolescent birth cohort.

Journal of Abnormal Psychology, 105, 299-312.

Lubinski, D., & Humphreys, L. G. (1997). Incorporating general intelligence into epidemiology and the social sciences. *Intelligence*, 24, 159-201.

Milne BJ, Byun U, Lee A. New Zealand Socioeconomic Index 2006. Wellington: Statistics New Zealand; 2013.

Muthén LK, Muthén BO. *Mplus User's Guide. Eighth Edition.* Los Angeles, CA: Muthén & Muthén (1998-2020).

Poulton, R., Moffitt, T. E. & Silva, P. A. (2015). The Dunedin Multidisciplinary Health and Development Study: overview of the first 40 years, with an eye to the future. Social Psychiatry and Psychiatric Epidemiology, 50, 679–693.

Richmond-Rakerd, L. S. et al. (2020). Clustering of health, crime and social-welfare inequality in 4 million citizens from two nations. Nature Human Behaviour, 44, 255–264.

Richmond-Rakerd LS, Caspi A, Ambler A, d'Arbeloff T, de Bruine M, Elliott M, Harrington HL, Hogan S, Houts RM, Ireland D, Keenan R, Knodt AR, Melzer TR, Park S, Poulton R, Ramrakha S, Rasmussen LJH, Sack E, Schmidt AT, Sison ML, Wertz J, Hariri AR, Moffitt TE. (2021). Childhood self-control forecasts the pace of midlife aging and preparedness for old age. PNAS, 118, 1-11.

Reuben A. Moffitt T.E. Caspi A. Belsky D.W. Harrington H. Schroeder F. et al. (2016). Lest we forget: Comparing retrospective and prospective assessments of adverse childhood experiences in the prediction of adult health. *Journal of Child Psychology and Psychiatry,* 57, 1103-1112.

Robins LN, Cottler L, Bucholz KK, Compton W. *Diagnostic Interview Schedule for DSM-IV*. St Louis, MO: Washington University School of Medicine; 1995.

Robins LN, Helzer JE, Croughan J, Ratcliff KS. (1981). National Institute of Mental Health Diagnostic Interview Schedule: Its history, characteristics, and validity. *Archives of General Psychiatry*, 38, 381-389.

Scottish Council for Research in Education. The Burt Word Reading Test, 1974 Revision. London, England: Hodder & Stoughton; 1976.

Stanton WR, Nada-Raja S, Langley J. (1995). Stability in the structure of health locus of control among adolescents. British Journal of Clinical Psychology, 34,132-140.

Wechsler D. (1974). *Manual for the Wechsler Intelligence Scale for Children – Revised*. New York, NY: Psychological Corporation.

Wechsler D. (2008). Wechsler Adult Intelligence Scale. 4th ed. San Antonio, TX: Pearson Assessment.

Wertz J., Israel S, Arseneault L, Belsky DW, Bourassa KJ, Harrington HL, Houts R, Poulton R, Richmond-Rakerd LS, Rosyamb, E, Moffitt TE, Caspi, A. (2021). Vital personality scores and healthy aging: Life-course associations and familial transmission. Social Science & Medicine, 285, 114283.
